# Adipo-glial signaling mediates metabolic adaptation in peripheral nerve regeneration

**DOI:** 10.1016/j.cmet.2023.10.017

**Published:** 2023-12-05

**Authors:** Venkat Krishnan Sundaram, Vlad Schütza, Nele H. Schröter, Aline Backhaus, Annika Bilsing, Lisa Joneck, Anna Seelbach, Clara Mutschler, Jose A. Gomez-Sanchez, Erik Schäffner, Eva Ernst Sánchez, Dagmar Akkermann, Christina Paul, Nancy Schwagarus, Silvana Müller, Angela Odle, Gwen Childs, David Ewers, Theresa Kungl, Maren Sitte, Gabriela Salinas, Michael W. Sereda, Klaus-Armin Nave, Markus H. Schwab, Mario Ost, Peter Arthur-Farraj, Ruth M. Stassart, Robert Fledrich

**Affiliations:** 1 Institute of Anatomy, Leipzig University, Leipzig, Germany; 2 Paul Flechsig Institute of Neuropathology, University Clinic Leipzig, Leipzig, Germany; 3 John Van Geest Centre for Brain Repair, Department of Clinical Neurosciences, University of Cambridge, Cambridge CB2 0PY, UK; 4 Instituto de Investigación Sanitaria y Biomédica de Alicante (ISABIAL), Alicante, Spain; 5 Instituto de Neurociencias CSIC-UMH, San Juan de Alicante, Spain; 6 Department of Neurobiology and Developmental Sciences, University of Arkansas for Medical Sciences, Markham, AR, USA; 7 Max Planck Institute of Experimental Medicine, Göttingen, Germany; 8 Klinik für Neurologie, Universitätsmedizin Göttingen (UMG), Göttingen, Germany; 9 NGS-Integrative Genomics Core Unit (NIG), Institute of Human Genetics, University Medical Center Göttingen, Göttingen, Germany

**Keywords:** peripheral nerve injury, Schwann cell, nerve repair, remyelination, regeneration, adipocytes, leptin, leptin receptor, myelin autophagy, myelinophagy, mitochondrial respiration, oxidative phosphorylation, energy metabolism, metabolic adaptation

## Abstract

The peripheral nervous system harbors a remarkable potential to regenerate after acute nerve trauma. Full functional recovery, however, is rare and critically depends on peripheral nerve Schwann cells that orchestrate breakdown and resynthesis of myelin and, at the same time, support axonal regrowth. How Schwann cells meet the high metabolic demand required for nerve repair remains poorly understood. We here report that nerve injury induces adipocyte to glial signaling and identify the adipokine leptin as an upstream regulator of glial metabolic adaptation in regeneration. Signal integration by leptin receptors in Schwann cells ensures efficient peripheral nerve repair by adjusting injury-specific catabolic processes in regenerating nerves, including myelin autophagy and mitochondrial respiration. Our findings propose a model according to which acute nerve injury triggers a therapeutically targetable intercellular crosstalk that modulates glial metabolism to provide sufficient energy for successful nerve repair.

## Highlights

• Nerve repair after acute trauma involves Schwann cell leptin receptor signaling
• Leptin receptor activation promotes glial metabolic adaptation post injury
• Schwann cell leptin receptor signaling is activated by adipose tissue-derived leptin

## Graphical abstract

**Figure:**
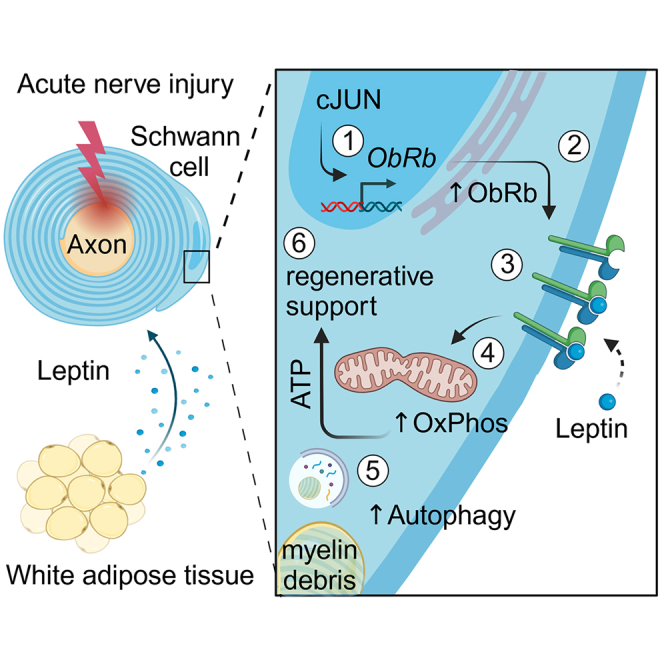

## Teaser

Peripheral nerves can regenerate after trauma, but full recovery is rare and critically relies on repair by Schwann cells. Here, Sundaram, Schütza, Schröter, et al. reveal that, after a nerve injury, adipocyte-derived leptin supports peripheral nerve repair. Leptin receptor signaling in Schwann cells adjusts catabolic processes, including mitochondrial respiration and autophagy, facilitating nerve repair.

## Introduction

Restoration of injured tissue is a fundamental biological process that has evolved to secure survival. The regenerative capabilities, however, can vary widely, depending on species, type of tissue, and context of injury.^1^ In mammals, the peripheral nervous system is a prime example of a tissue with regenerative potential. The long axons that connect the central nervous system with the periphery closely interact with highly plastic glia, the Schwann cells, which wrap large caliber axons with electrically insulating myelin to facilitate rapid impulse propagation.^2^ Following injury, the axons distal to the injury site undergo Wallerian degeneration, leaving the nerve with axonal remnants and growth inhibiting myelin debris that must be cleared prior to regeneration.^3^ Importantly, Schwann cells in the injured nerve undergo adaptive cellular reprogramming into repair cells,^4^ which orchestrate debris clearance by myelin autophagy,^5^ phagocytosis,^6^ and attraction of macrophages.^7^ Repair Schwann cells then guide and trophically support regrowing axons and eventually reconstitute myelin.^8^ Although remarkably efficient under idealized experimental conditions, any perturbation of this repair program impacts functional nerve regeneration. Indeed, the clinical outcome of nerve injuries usually remains poor and nerve damage constitutes a significant clinical and economic burden.^9^^,^^10^

In general, the energy-demanding nature of successful tissue repair requires vital local metabolic adaptations.^11^ Acute nerve injury indeed poses a comprehensive metabolic challenge toward Schwann cells, which need to employ both catabolic and anabolic cellular processes to accomplish nerve de- and regeneration. Although Schwann cells have been shown to acutely respond to injury by glycolytic activation,^12^ the dynamics of metabolic homeostasis during nerve repair and its regulatory mechanisms in regenerating Schwann cells remain unknown so far.

Here, we show that acute nerve injury provokes a pronounced metabolic adaptation in Schwann cells, with an induction of mitochondrial oxidative phosphorylation (OxPhos) throughout the time course of nerve repair. As an upstream regulator, we identified leptin receptor signaling in Schwann cells to modulate glial metabolism in nerve regeneration. The integration of adipocyte-derived leptin by Schwann cells promotes myelin autophagy and glial OxPhos in the course of nerve repair and remyelination. These discoveries suggest that adipocytes and Schwann cells interact to regulate metabolic homeostasis after nerve injury, which may open new treatment strategies for nerve trauma and beyond.

## Results

### Schwann cells display a mitochondrial response in injured nerves

**Figure 1:**
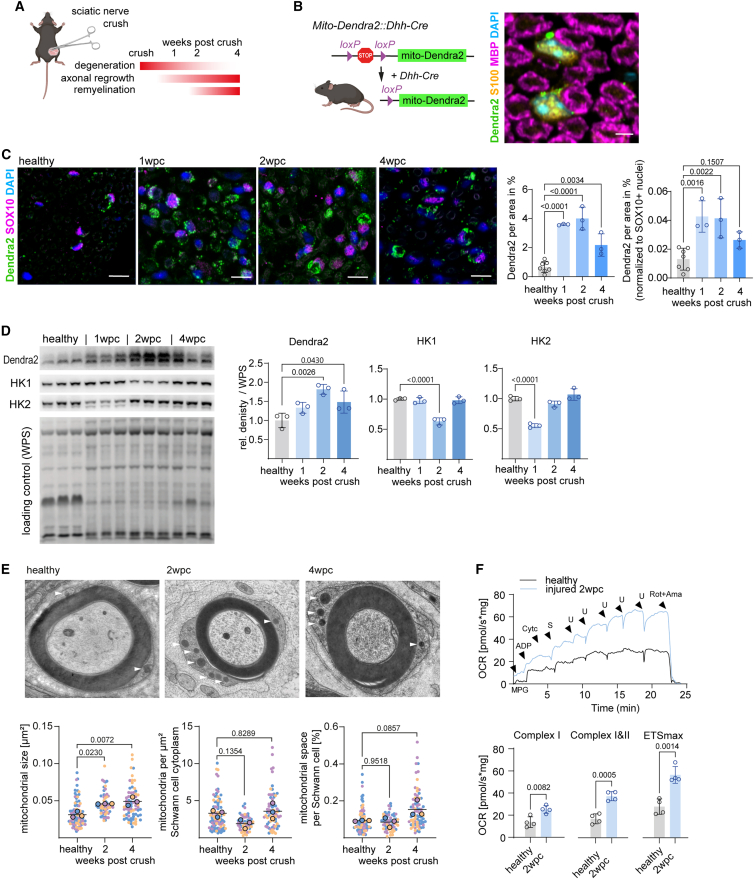
Schwann cells upregulate mitochondrial respiration after peripheral nerve injury (A) Experimental strategy for nerve crush surgeries. After unilateral crush surgery at the proximal sciatic nerve, the distal sciatic nerve is collected at indicated time points. At 1 week post nerve crush (1 wpc), the distal nerve is in a peak degenerative phase, whereas axonal regrowth is about to start. At 2 wpc, degeneration is still present, but axonal regrowth has progressed and remyelination is initiated. At 4 wpc, axonal regeneration and remyelination are largely completed. (B) Schematic representation of the genetic strategy to generate Schwann cell-specific mitochondria reporter mice (left). Schwann cell mitochondria are labeled by Dendra2 (green), Schwann cell cytoplasm by S100 (orange), myelin by MBP (magenta), and nuclei by 4',6-diamidino-2-phenylindole (DAPI) (blue). (C) Sciatic nerve cross sections of mice from (B), healthy, 1, 2, and 4 wpc (left, Schwann cell mitochondrial Dendra2 in green, Schwann cell nuclei SOX10 in magenta, and all nuclei DAPI in blue; scale bars, 10 μm). The Dendra2 area (in percent) is normalized to the total area of the sections (left quantification) and to SOX10-postive Schwann cell numbers (right quantification). (D) Western blot analyses and Dendra2 quantification of distal sciatic nerve endoneurial protein lysates from reporter mice from (B) revealed an increase at 1, 2, and 4 wpc. Hexokinase 1 and 2 (HK1 and HK2) show a reduction at the indicated time points. Quantification was performed relative to whole protein stain (WPS), which was identified as the most stable loading control (Figure S1A, one-way ANOVA with Dunnett’s post test). (E) Electron microscopic assessment of Schwann cell mitochondria in distal sciatic nerves at 2 and 4 wpc for mitochondrial size, number (normalized to Schwann cell cytoplasmic area), and space occupancy in Schwann cells. Representative electron micrographs of nerve cross sections are shown on top and the quantifications below. Arrowheads point to mitochondria. Data are derived from *Lepr*-ctrl mice (see Figure 3A and re-used in Figure 4D [nested one-way ANOVA with post test]). (F) Representative respirometric traces of the oxygen consumption rate (OCR) in sciatic nerve endoneuria from contra- (healthy) and ipsilateral (injured) sites of adult wild-type mice at 2 wpc. Traces represent the oxygen flux per sciatic nerve mass in mg (top panel). Quantification shown in bottom panel for complex I substrates, complex I and II, and maximal electron transfer system (ETS_max_) (n = 4 per group, unpaired t tests; M, malate; P, pyruvate; G, glutamate; S, succinate; Cyt*c*, cytochrome *c*; U, chemical uncoupler/CCCP, carbonyl cyanide m-chlorophenyl hydrazone; Rot, rotenone; Ama, antimycin A).

In order to analyze the energy metabolic response of Schwann cells during peripheral nerve regeneration, we employed an experimental nerve crush model (Figure 1A). This model allows us to follow Wallerian degeneration, axonal regrowth, and remyelination along with the respective repair response of Schwann cells during a well-defined standardized time course of 4 weeks (Figure 1A). Immunohistochemical and western blot analyses of whole endoneurial tissue lysates after sciatic nerve crush revealed an increased expression of all five complexes of the respiratory chain (Figures S1A and S1B), reflecting all endoneurial cells, including Schwann cells, axons, and macrophages (Figure S1C). In order to be able to specifically investigate Schwann cell mitochondria after sciatic nerve crush, we generated and took advantage of conditional Schwann cell-specific reporter mice that express the mitochondrial membrane-targeted fluorescent protein Dendra2 (Figure 1B).^13^^,^^14^ Notably, immunohistochemical and western blot analyses with injured sciatic nerves from glial Dendra2 reporter mice revealed a pronounced mitochondrial expansion in Schwann cells throughout peripheral nerve regeneration, with a peak at 2 weeks post nerve crush (2 wpc; Figures 1C and 1D). In contrast, a respective analysis of mitochondrial expansion in spinal motoneuron-derived axons via Chat-Cre-driven Dendra2 fluorescent reporter mice showed much lower baseline abundance and only a mild, non-significant induction of Dendra2 expression after nerve injury (Figures S1D–S1F). To further investigate the mitochondrial response in Schwann cells after nerve injury, we performed electron microscopy and mitochondrial morphometry, where we found an increase in mitochondrial size at 2 and 4 weeks after injury (Figure 1E). We next applied *ex vivo* high-resolution respirometry, which allows us to measure mitochondrial respiration within the complex endoneurial tissue environment after nerve injury. Importantly, we found here the mitochondrial expansion in Schwann cells and possibly other endoneurial cells to be associated with a strong increase in the oxygen consumption rate (OCR) within the endoneurial compartment of injured sciatic nerves (Figure 1F).

**Figure 2:**
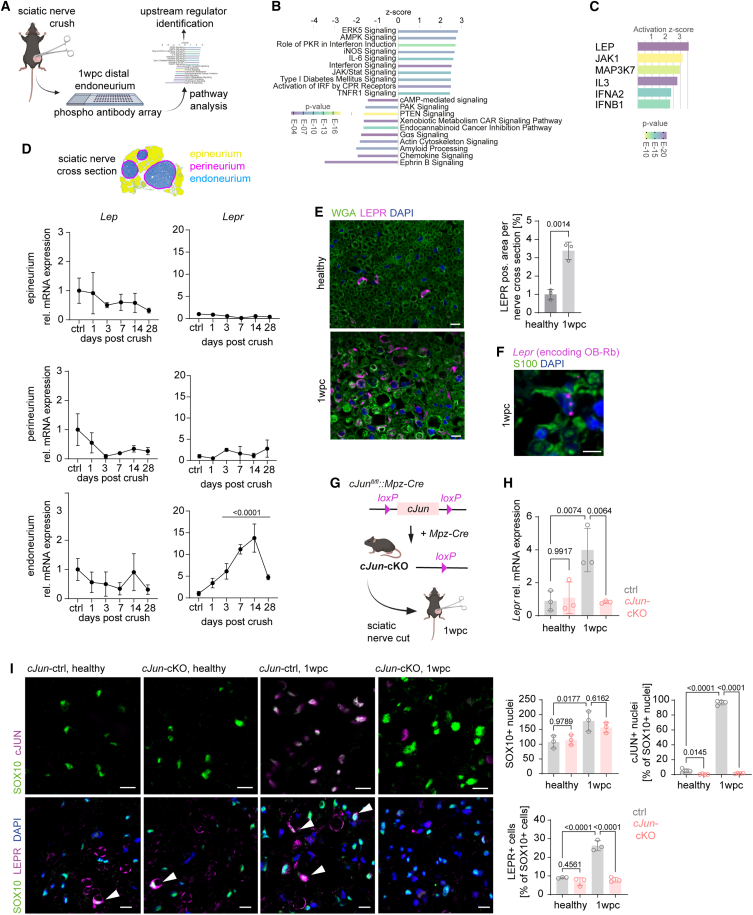
Leptin signaling coincides with the oxidative shift in injured peripheral nerves (A) Strategy to identify upstream regulators of metabolic adaptation in Schwann cells of injured nerves employing phospho-protein explorer arrays. (B) Identification of regulated pathways in injured nerves as retrieved from (A). Shown is the ranking of the top 20 inversely regulated pathways between ipsi- and contralateral sciatic nerve endoneuria sorted by activation *Z* score (ingenuity pathway analysis [IPA] comparison analysis). (C) Identified putative upstream regulators of regulated pathways after nerve injury. Ranking of the top six upstream regulators sorted by activation *Z* score revealed leptin signaling as the strongest candidate. Candidates were determined by IPA upstream analysis based on the comparison analysis from (B) (p value cutoff: p < 10^−10^). (D) Analyses of mRNA expression of leptin (*Lep*) and leptin receptor (*Lepr*) genes in different compartments of the sciatic nerve (epineuria, yellow; perineuria, pink; endoneuria, blue in schematic representation of a sciatic nerve cross section) from adult wild-type mice across different days post crush (n = 3–5 per group, expression normalized to ctrl levels, one-way ANOVA with Dunnett’s post test). (E) Immunohistochemical stainings and quantification of LEPR in tibial nerve cross sections from contra- (healthy) and ipsilateral (injured 1 wpc) sites of adult wild-type mice. Plasma membranes (wheat germ agglutinin [WGA], green), LEPR (magenta), and cell nuclei (DAPI, blue) are shown in the representative images (scale bars, 10 μm; n = 3 per group, Student’s t test). (F) BaseScope *in situ* hybridization for *Lepr* mRNA encoding the long isoform of the leptin receptor, Ob-Rb (magenta), on a tibial nerve cross section at 1 wpc. Schwann cell cytoplasm (S100, green) and cell nuclei (DAPI, blue; scale bars, 5 μm). (G) Schematic representation of the genetic strategy and experimental plan for Schwann cell-specific *cJun* conditional knockout mice (*cJun-*cKO). (H) *Lepr* mRNA expression at 1 wpc in healthy and injured tibial nerves of ctrl and *cJun-*cKO mice (standardized to *Ankrd27* and *Canx*, one-way ANOVA with Tukey’s post hoc test). (I) Immunohistochemistry in healthy and injured (1 wpc) nerves of control and *cJun*-cKO mice against SOX10 (green) and cJUN (magenta, top row) and against SOX10 (green) and LEPR (magenta, bottom row, one-way ANOVA with Tukey’s post hoc test; scale bars, 10 μm).

What regulates the induction of mitochondrial metabolism after acute nerve injury? To identify potential upstream regulators, we took advantage of phospho-protein explorer antibody arrays (Figure 2A), which showed a pronounced induction of numerous signaling pathways upon nerve injury, including pathways implicated in the regulation of cellular metabolism (Figure 2B). Interestingly, an upstream activator analysis revealed leptin as a putative regulator of this signaling response in Schwann cells at 1 wpc (Figure 2C), i.e., at a time point when Schwann cells perform myelin autophagy and provide a permissive environment for axonal regeneration.^6^,^5^,^8^ Notably, leptin is a circulating cytokine released from adipocytes and is best known for its role in regulating appetite and satiety in the hypothalamus.^15^ However, the leptin receptor has been shown to mediate metabolic processes in a variety of tissues,^16^ but a regulatory function in Schwann cell metabolism has not been described thus far.

In order to assess a potential role of leptin signaling in the injured peripheral nerve, we first performed transcriptional analyses of leptin and the leptin receptor in different subcompartments of the sciatic nerve distal to the injury site throughout the time course of nerve de- and regeneration (Figure 2D). Here, we identified a strong, endoneurium-specific induction of the leptin receptor gene *Lepr* from day 3 post crush that lasts throughout the time course of de- and regeneration, with a peak at 14 days post nerve crush (Figure 2D). Importantly, we could attribute the strong leptin receptor protein expression in endoneuria after injury to Schwann cells by immunohistochemistry (Figure 2E), using an antibody that we validated in leptin receptor mutant *db*/*db* mice (Figure S2A). Notably, the leptin receptor gene gives rise to a variety of isoforms^17^ of which only the long Ob-Rb isoform harbors the entire intracellular domain with binding sites for downstream signaling molecules.^18^ Thus, next to isoform-specific qPCR (Figure 2D), we employed specific BaseScope *in situ* hybridization to confirm the upregulation of *Lepr* transcripts that give rise to the long Ob-Rb isoform (Figure 2F). In general, the repair response of Schwann cells is characterized by an induction of the expression of the transcription factor cJun.^19^ Notably, the specific ablation of cJun from Schwann cells strongly delays nerve degeneration and severely impairs regeneration after acute nerve injury.^19^ We therefore next investigated if induction of *Lepr* in Schwann cells is dependent on cJun expression after injury by taking advantage of conditional mouse mutants that lack cJun expression in Schwann cells (*cJun*-cKO; Figures 2G–2I). We first confirmed that after nerve injury, almost all Schwann cells become positive for cJUN expression (Figure 2I). Importantly, conditional ablation of cJUN from Schwann cells in *cJun*-cKO mice resulted in an abolished induction of both *Lepr* mRNA, as well as of LEPR protein 1 wpc (Figures 2H and 2I). Hence, Schwann cells after nerve injury require cJUN to induce *Lepr* expression in the course of nerve repair.

### Leptin receptor signaling in Schwann cells promotes nerve regeneration

**Figure 3:**
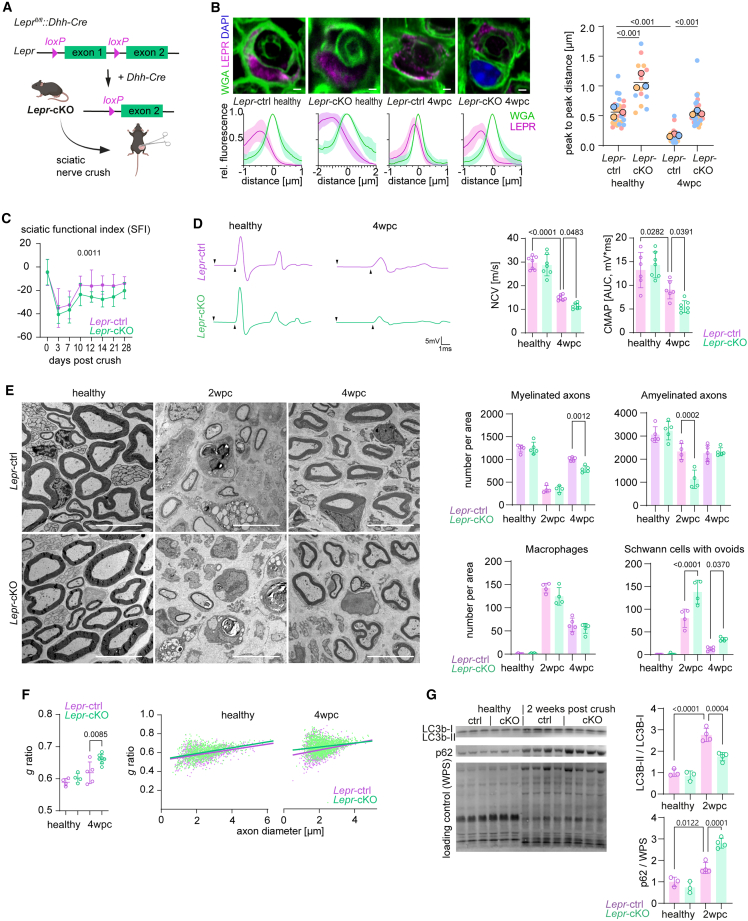
Ablation of *Lepr* from Schwann cells impairs regeneration after acute nerve injury (A) Schematic representation of the genetic strategy and experimental plan for conditional *Lepr* knockout mice. (B) Immunohistochemical stainings of tibial nerve cross sections (upper left panels) of contra- (healthy) and ipsilateral (4 wpc) sites from adult control (*Lepr*-ctrl) and Schwann cell *Lepr* knockout mice (*Lepr*-cKO). Plasma membranes (WGA, green), leptin receptor protein (LEPR, magenta), and cell nuclei (DAPI, blue) are depicted (scale bars, 2 μm). Cumulative fluorescence intensities of WGA and LEPR stainings (lower left panels) were assessed across the cell membrane (bottom). Quantification (right, group mean with SD; mean per animal [large circles] and individual data points [small circles] are shown) was performed by determining peak-to-peak distances between WGA and LEPR fluorescent signals (n = 3 per group, nested one-way nested ANOVA with Tukey’s post hoc test). (C) Impaired functional recovery of *Lepr*-cKO mice after injury as revealed by DigiGait walking analysis and calculation of the sciatic nerve functional index (SFI) of adult control (*Lepr*-ctrl, magenta) and Schwann cell *Lepr* knockout mice (*Lepr*-cKO, green, n = 7 per group, two-way ANOVA). (D) Electrophysiological recordings of healthy and injured (4 wpc) *Lepr*-cKO (green) mice compared with controls (magenta) after nerve injury. Representative electroneurographic traces (left) and calculation of the nerve conduction velocity (NCV) and compound muscle action potential (CMAP, as area under the curve, right panels) are shown (n = 6–7 per group, one-way ANOVA with Tukey’s post hoc test). (E) Representative electron micrographs (left; scale bars, 10 μm) of ultra-thin tibial nerve cross sections from adult control (*Lepr*-ctrl) and Schwann cell *Lepr* knockout (*Lepr*-cKO) mice before (healthy) and 2 and 4 weeks after crush (2 and 4 wpc). Quantifications (right) of the number of amyelinated and myelinated axons, macrophages, and Schwann cells that contain myelin degeneration profiles was performed and expressed per cross sectional area (21.536 μm², n = 4–7 per group, one-way ANOVA with Tukey’s post hoc test). (F) Analysis of myelin sheath thickness by calculation of the *g*-ratio from data in (E) (n = 4–7 per group, one-way ANOVA with Sidak’s post hoc test). (G) Western blot analysis (left) and quantification (right) in sciatic nerve endoneurium lysates of contra- (healthy) and ipsilateral sites from control (*Lepr*-ctrl, magenta) and Schwann cell *Lepr* knockout (*Lepr*-cKO, green) mice at 2 wpc. The ratio of LC3b-II over LC3b-I was calculated, for the p62 protein abundance was normalized to WPS as loading control, n = 3–4 per group, one-way ANOVA with Tukey’s post hoc test.

Is the increased leptin receptor expression implicated in the metabolic response of Schwann cells after injury and functionally relevant for nerve repair? To address this question, we generated mutant mice that lack exon 1 of the *Lepr* gene selectively in Schwann cells during embryogenesis (*Lepr*^*fl/fl*^*::Dhh-Cre, Lepr*-cKO; Figures 3A and S2C). These mice still express a truncated version of the leptin receptor, without the signal peptide, preventing its translocation and exposure on the cellular surface.^20^^,^^21^ In line with this, we detected an increased Schwann cell plasma membrane presentation of the leptin receptor in control mice after injury (Figure 3B), including dynamic regulation of *Leprot* and *Rnf41* (Figure S2B), two genes for proteins known to regulate LEPR trafficking.^16^ Of note, LEPR membrane translocation could be observed in Schwann cells of control but not *Lepr-*cKO mice, confirming the validity of the genetic approach (Figure 3B). Importantly, *Lepr*-cKO mice showed no alterations in peripheral nerve development or in the non-injured adult peripheral nerve (Figures 3D–3F), allowing to investigate a specific function of leptin receptor signaling in Schwann cells during nerve regeneration. We hence applied an experimental nerve injury to *Lepr*-cKO mice and first tracked functional nerve repair by digital gait analysis, which we found to be impaired in *Lepr*-cKO mice compared with injured controls during the course of nerve regeneration (Figure 3C). In line with this, electrophysiological recordings at 4 wpc (when nerve repair is completed in wild-type [WT] animals) revealed decreased compound muscle action potentials (CMAPs), indicative of a reduced number of functionally regenerated axons in *Lepr*-cKO mice (Figure 3D). Furthermore, nerve conduction velocity (NCV), a surrogate marker of remyelination, was significantly decreased in *Lepr*-cKO mice compared with control mice at 4 wpc (Figure 3D). In order to correlate these findings to nerve histopathology, we next performed electron microscopy at 2 and 4 wpc and quantified axonal regrowth and remyelination. Here, we detected a transiently decreased number of total axons in *Lepr*-cKO mutants (Figure S2D) (along with a respective reduction of amyelinated, but not myelinated, fibers at 2 wpc; Figure 3E), indicating an impaired initial axonal regeneration. Notably, although the total number of regenerating axons recovered at 4 wpc (Figure S2D), we observed a reduced number of myelinated fibers, as well as thinner myelin sheaths in *Lepr*-cKO compared with injured controls at 4 wpc (Figures 3E and 3F). Hence, we conclude that *Lepr*-cKO mice first suffer from a transient delay in axonal regeneration, which is followed by an impaired remyelination of newly regrown fibers.

In order to investigate the mechanisms underlying impaired nerve repair in *Lepr*-cKO mice, we analyzed macrophage abundance and Schwann cell characteristics post nerve injury. Here, we did not detect a difference in the number of macrophages (Figures 3E and S2E) but transient differences in Schwann cell numbers in the regenerating nerve (at 2 wpc; Figures S2D and S2F), along with a reduced rate in Schwann cell proliferation at the same time point (Figure S2F). The expression of genes involved in adaptive cellular reprogramming or redifferentiation in Schwann cells after injury, however, was not altered in *Lepr*-cKO mice compared with respective controls (Figure S2I).

However, we observed a delay in myelin debris removal in *Lepr*-cKO mice, reflected by a higher number of Schwann cells with myelin ovoids (myelin degeneration profiles) in *Lepr*-cKO mutants compared with controls (Figure 3E). Do Schwann cells in *Lepr*-cKO suffer from an impaired myelin autophagy during peripheral nerve repair? Notably, myelin autophagy was strongly induced in WT animals at 2 and 4 weeks after peripheral nerve injury (Figures 3G and S2J). In contrast, *Lepr*-cKO mice displayed a reduced autophagic activity in whole endoneurial lysates at both time points post nerve crush, reflected by a reduced lipidation of LC3b-I to LC3b-II, and an accumulation of the autophagy substrate p62 at 2 wpc (Figures 3G and S2J). Importantly, it was previously shown that disruption of autophagy by *Atg7* ablation in Schwann cells results in a delay of nerve regeneration, but does not ultimately impair nerve repair or remyelination at 4 wpc.^22^ Our finding of impaired remyelination in *Lepr*-cKO mice at 4 wpc may therefore hint at a more generalized function of leptin receptor signaling beyond myelinophagy in nerve repair.

### Leptin receptor signaling modulates mitochondrial respiration during regeneration

**Figure 4:**
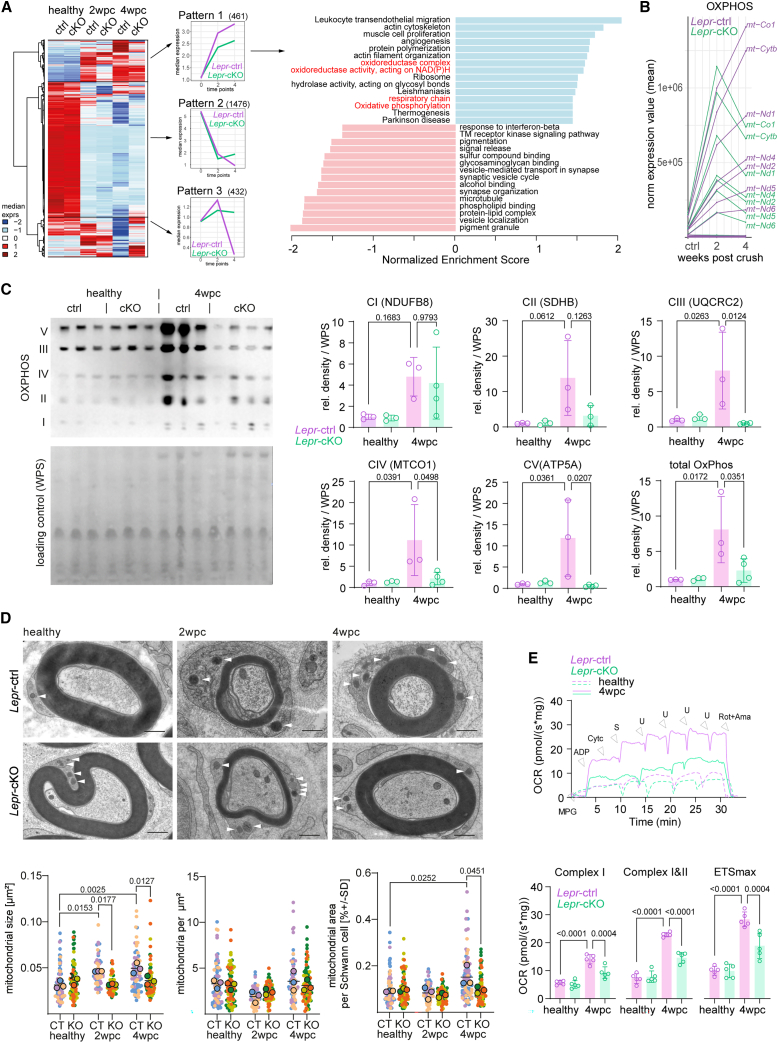
Leptin receptor signaling induces mitochondrial respiration in remyelinating Schwann cells (A) Bulk RNA sequencing of healthy and injured nerves from control (ctrl) and *Lepr*-cKO (cKO) mice with heat map of differentially expressed genes (left). Three patterns were derived from differential gene expression analysis at 2 and 4 wpc. First, genes up in ctrl but downregulated in cKO at 2 and 4 wpc (pattern 1); second, genes downregulated in cKO at 2 wpc and upregulated at 4 wpc (pattern 2); and third, genes upregulated in cKO at 2 but downregulated at 4 wpc (pattern 3). Top 30 dysregulated processes from pattern 1 (right) were ranked according to the normalized enrichment score (NES) using WebGestaltR (version 0.4.4) for Reactome pathways (n = 4 per group). (B) Temporal expression patterns between *Lepr*-ctrl (magenta) and *Lepr*-cKO (green) are shown. Solid lines connect the average expression of individual genes associated with the mitochondrial gene set for oxidative phosphorylation (OxPhos, as extracted from MitoCarta). (C) Western blot analyses (left) and quantifications (right) of oxidative phosphorylation protein complexes I–V (OxPhos I–V) protein abundances in sciatic nerve endoneurium lysates of contra- (healthy) and ipsilateral (4 wpc) sites from adult control (ctrl) and Schwann cell *Lepr* knockout (cKO) mice (n = 3–4 per group, one-way ANOVA with Tukey’s post hoc test; WPS used as loading control). (D) Representative electron micrographs of tibial nerve cross sections from contra- (healthy) and ipsilateral (2 and 4 wpc) sites of adult control (*Lepr*-ctrl, CT) and Schwann cell *Lepr* knockout (*Lepr*-cKO, KO) mice at 2 and 4 wpc (top; scale bars, 1 μm). Mitochondria per area, mitochondrial size, and the relative occupancy of mitochondria per Schwann cell cytoplasmic area were quantified (bottom). Data of control samples (Lepr-ctrl) were re-used in Figure 1E (n = 3–5 per group; mean ± SD per group; individual means per animal [large circles] and mitochondrial means per Schwann cell [small circles]; nested one-way ANOVA with Tukey’s post hoc test). (E) Representative respirometric traces of the oxygen consumption rate (OCR) in sciatic nerve endoneuria from healthy and injured (4 wpc) sites of control and *Lepr*-cKO mice at 4 wpc. Traces represent the oxygen flux per sciatic nerve mass in mg (top). Quantification shown in bottom panel for complex I substrates, complex I and II, and maximal electron transfer system (ETS_max_) (n = 5 per group, one-way ANOVA and Holm-Šidák multiple comparisons; M, malate; P, pyruvate; G, glutamate; S, succinate; Cyt*c*, cytochrome *c*; U, chemical uncoupler/CCCP, carbonyl cyanide m-chlorophenyl hydrazone; Rot, rotenone; Ama, antimycin A).

To identify additional functions of leptin receptor signaling in repair Schwann cells, we next analyzed known leptin receptor downstream signaling pathways at the western blot level, which revealed an impaired activation of PI3K/AKT, MEK/ERK, and JAK/STAT in *Lepr*-cKO mice compared with controls after nerve injury (Figure S3). Most differences in the ratios between the phosphorylated protein over the constitutive protein, however, are the consequence of increased expression of the constitutive protein resulting in reduced ratios, potentially due to secondary mechanisms after *Lepr* ablation. As western blot analyses of full endoneurial lysates do not allow us to resolve the cellular source, we performed immunohistochemistry for pSTAT3, where we found the number of pSTAT3-positive Schwann cells in *Lepr*-cKO mutants to be reduced when compared with controls at 4 wpc, indicating an influence of leptin receptor signaling on the JAK/STAT signaling pathway at later phases of nerve repair (Figure S4A). We next performed an unbiased bulk RNA sequencing (RNA-seq) in *Lepr*-cKO endoneuria at 2 and 4 wpc, which revealed more than 2,300 differentially expressed genes that cluster according to different trajectories of temporal regulation after nerve injury (Figure 4A). Enrichment analysis revealed, among others, genes related to mitochondrial respiration to be strongly upregulated and maintained during nerve regeneration in control but not in *Lepr*-cKO mice (Figure 4A). A respective visualization of the regulation of all genes involved in OxPhos, which we retrieved from MitoCarta,^23^ highlighted an induction of this gene set in both control and *Lepr*-cKO mice at 2 wpc. However, *Lepr*-cKO mice appeared unable to maintain the expression of mitochondria-related genes during the time course of nerve regeneration, in contrast to control mice (Figure 4B). We hence next tested protein expression of OxPhos components by western blotting and also found a strongly reduced induction of OxPhos protein expression at 4 wpc in full endoneurial lysates of *Lepr*-cKO mice compared with injured controls, which reached significance for the complexes III, IV, and V of the tested antibody cocktail (Figure 4C). Of note, RNA-seq and western blot analyses were performed on whole endoneurial lysates and contain information of all endoneurial cell types. Therefore, potential subtle changes in mitochondrial gene expression in Schwann cells of *Lepr*-cKO mice at 2 wpc may be masked by expression patterns of other cell types. However, we hypothesized that changes in mitochondrial gene and protein expression in *Lepr*-cKO mice results from Schwann cells and, indeed, we found OxPhos protein expression in Schwann cells to be significantly reduced in *Lepr*-cKO mice compared with controls at 4 wpc when assessed via immunohistochemistry (Figure S2G). Does this lower OxPhos expression correlate with mitochondrial morphology that we found to increase in size upon injury (Figure 1E)? To address this question, we again analyzed Schwann cell mitochondria by electron microscopy of cross sections from injured nerves. Here, we detected no changes in mitochondria numbers (when normalized to Schwann cell cytoplasmic area); however, we found the injury-induced increase in mitochondria size and relative space occupancy within Schwann cells to be abolished in *Lepr*-cKO mice at 2 and 4 wpc (Figure 4D). Importantly, axonal mitochondria were not visibly altered upon injury or in *Lepr*-cKO (Figure S2H). When we next addressed the functional consequences of impaired mitochondrial adaptations in *Lepr*-cKO mice by measuring mitochondrial respiration in whole endoneurial tissue *ex vivo*, we observed a strongly reduced OCR in *Lepr*-cKO endoneuria at 4 wpc when compared with controls (Figure 4E). Together, these data suggest a model in which leptin receptor signaling drives myelin autophagy and an injury-related mitochondrial metabolic response that is associated with optimal nerve regeneration.

**Figure 5:**
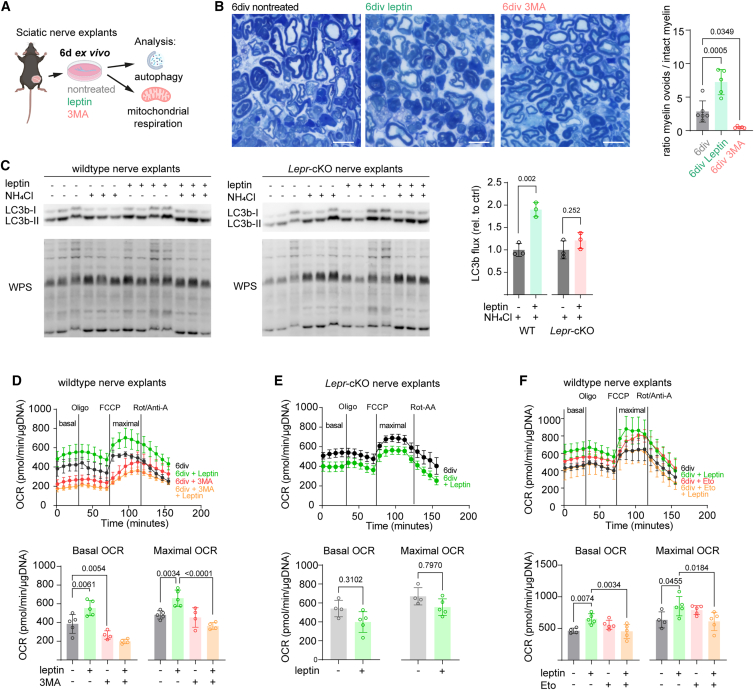
Leptin promotes autophagy and mitochondrial respiration *ex vivo* (A) Schematic presentation of the experimental setup to investigate the impact of leptin on nerve metabolism in *ex vivo* sciatic nerve cultures. (B) Histological assessment of nerve explant semi-thin cross sections after 6 days *ex vivo* (6 div) and quantification of the ratio of degenerative myelin profiles (ovoids) over intact myelin structures in leptin-treated (green) compared with nontreated (gray) nerves. Nerve explants treated with 3-MA (red) displayed almost no myelin degeneration. Representative semi-thin images are shown on the left (scale bars, 10 μm), quantification on the right (n = 5–6 nerves per group, one-way ANOVA with Tukey’s post test). (C) Western blot analysis of LC3B autophagic flux in nerve explants upon leptin treatment in WT (left) and *Lepr*-cKO nerves (middle). Nerve explants were either left nontreated or treated with recombinant leptin or lysosomal blocker NH_4_Cl or both recombinant leptin and NH_4_Cl for 6 days. Quantifications are shown on the right (data expressed as fold change, n = 3 per group, Student’s t test). (D–F) Seahorse respirometry and measurement of the oxygen consumption rate (OCR) in WT (D and F) or *Lepr*-cKO (E) nerve explants after 6 days *ex vivo* (6 div). Explants were left untreated (black, D and E), treated with leptin (green, D–F), treated with 3-MA (red, D), treated with both leptin and 3-MA (orange, D), treated with the fatty acid oxidation inhibitor etomoxir (ETO, red, F), or treated with both leptin and etomoxir (orange, F). OCR traces are depicted on top, and quantification of the basal and maximal OCR is shown at the bottom (n = 4–5 per group, one-way ANOVA with Tukey’s post test).

Are these two catabolic processes, autophagy and mitochondrial respiration, coupled in injured Schwann cells? To investigate a potential link in more detail, we took advantage of cultured sciatic nerve explants, an established *ex vivo* model for nerve degeneration, which includes the whole endoneurial cellular landscape, including Schwann cells, fibroblasts, and resident macrophages, but excludes the aspect of additional invasion of humoral macrophages after nerve injury (Figure 5A).^5^ We first investigated if myelin and axon degeneration is affected by leptin. When recombinant leptin is added to the culture medium, the relative number of degenerating profiles (i.e., myelin ovoids) is increased, an effect that is completely blocked when autophagy is inhibited by 3-methyladenine (3-MA) (Figure 5B). Indeed, when assessing the autophagic flux in nerve explants by western blot, i.e., by the accumulation of LC3b-II upon inhibition of lysosomal fusion with ammonium chloride, we found the presence of leptin in the culture medium to increase the autophagic flux in nerves from WT, but not from Schwann cell-specific *Lepr*-cKO mice (Figure 5C), consistent with the *in vivo* findings in *Lepr*-cKO mice post nerve crush (Figure 3G). Likewise, when mitochondrial respiration in response to leptin treatment was analyzed by respirometry, both the basal and the maximal OCR were enhanced in leptin-treated versus nontreated *ex vivo* WT nerves (Figure 5D), but not in *Lepr-*cKO nerves (Figure 5E). To assess the dependence of mitochondrial OxPhos on autophagy activation in the injured nerve, we next applied an inhibitor of autophagy, 3-MA (Figure 5D). Importantly, inhibition of autophagy significantly reduced the basal, but not maximal, OCR in injured nerves 6 days *ex vivo* (Figure 5D). This indicates that mitochondrial respiration is coupled to autophagic activity in the injured nerve but that blocking autophagy does not reduce the maximal respiratory capacity. Notably, a concomitant leptin treatment in addition to 3-MA was not able to overcome impaired basal and did not gain maximal mitochondrial respiration (Figure 5D). We hence conclude that autophagy promotes OxPhos in the injured nerve and that leptin signaling may, at least in part, regulate mitochondrial respiration via autophagy in repair Schwann cells. This catabolic coupling between mitochondrial respiration and myelinophagy could be realized by oxidation of myelin lipids that would fuel OxPhos. To test this hypothesis, we treated nerve explants *ex vivo* with an inhibitor of fatty acid oxidation, etomoxir (ETO), and performed mitochondrial respirometry (Figure 5F). ETO treatment alone did not impair basal or maximal OxPhos, which may be explained by alternative substrate availability for mitochondrial respiration (in contrast to a complete blockage of autophagy), (Figure 5D). However, the increased respiratory capacity after leptin treatment was completely abolished when fatty acid oxidation was pharmacologically inhibited by ETO in *ex vivo* injured nerves (Figure 5F). Together, these data suggest that leptin modulates mitochondrial respiration in Schwann cells via autophagy and fatty acid oxidation after peripheral nerve injury. To investigate if the JAK/STAT3 pathway is implicated in leptin-mediated metabolic adaptation, we again treated *ex vivo* WT nerves with leptin and now co-applied the specific STAT3 inhibitor C188-9. We found STAT3 inhibition to reduce both autophagy (Figure S4B) and the OCR (Figure S4C) in leptin-treated nerves, suggesting involvement of JAK/STAT3 signaling in leptin-mediated metabolic adaptation in injured nerves. But what is the cellular source of leptin *in vivo* that activates leptin receptor signaling in Schwann cells during nerve regeneration?

### Adipocyte-derived leptin promotes nerve regeneration

**Figure 6:**
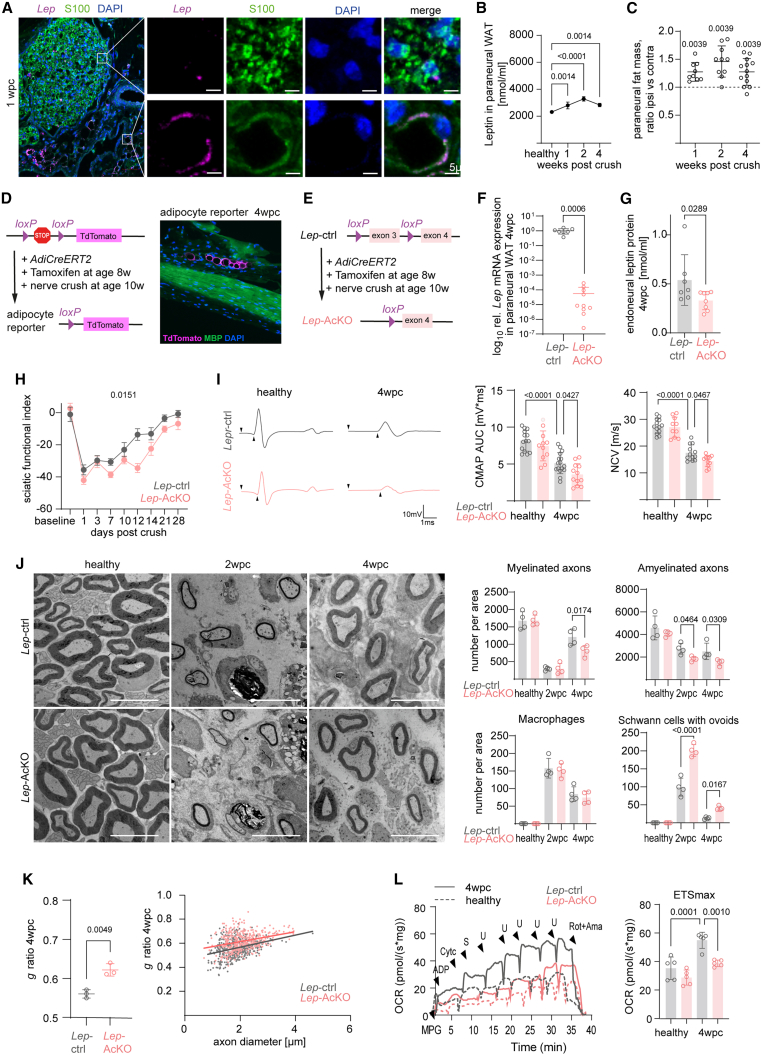
Adipocyte-derived leptin is required for efficient nerve repair (A) BaseScope *in situ* hybridization is shown with a *Lep* probe (magenta) on a tibial nerve cross section from an adult wild-type mouse 1 wpc. Schwann cells (S100) and cell nuclei (DAPI) are also depicted. Blow ups show leptin mRNA in a single Schwann cell (top) and an epineurial adipocyte (bottom, identified by morphology by autofluorescence in the S100 channel; scale bars, 5 μm). (B) ELISA quantification of total leptin protein in adult healthy and crushed wild-type mice in paraneural white adipose tissue (WAT) at 1, 2, and 4 wpc (n = 4–5, one-way ANOVA with Sidak’s post test). (C) Quantification of the paraneural fat mass after nerve injury at 1, 2, and 4 wpc. Quantification is shown as ratio of ipsi- versus contralateral fat mass per animal and individual data points display ratios per animal (n = 9–13 per time point, Wilcoxon matched-pairs signed rank test for each time point). (D) Schematic representation of the genetic strategy used for the generation and use of conditional adipocyte reporter mice (left). Representative immunohistochemical staining of a longitudinal sciatic nerve section from a recombined adult adipocyte reporter mouse 4 wpc depicts reporter-positive epineurial adipocytes (TdTomato, magenta) and reporter-free endoneurial myelin (MBP, green) and cell nuclei (DAPI, blue, right). (E) Schematic representation of the genetic strategy used for the generation and use of inducible conditional adipocyte *Lep* knockout mice. (F) Confirmation of recombination efficiency in paraneural WAT of *Lep-*AcKO mice at 4 weeks post nerve crush (4 wpc) by qPCR and measurement of relative *Lep* mRNA expression (n = 7–10, Student’s t test). (G) ELISA for leptin in endoneuria in *Lep*-ctrl (gray) and *Lep*-AcKO (rose) at 4 wpc (n = 7–8 per group, Student’s t test). (H) DigiGait walking analysis and calculation of the sciatic nerve functional index (SFI) of the injured side at different time points post sciatic nerve crush in *Lep*-ctrl (gray) and *Lep*-AcKO (rose) mice (n = 7 per group, two-way ANOVA). (I) Representative electrophysiological traces (left) and quantification of the compound muscle action potentials (CMAPs) and nerve conduction velocities (NCVs) of contra- (healthy) and ipsilateral (4 wpc) sites from *Lep*-ctrl and *Lep*-AcKO mice at 4 wpc (n = 12–16 per group, one-way ANOVA with Tukey’s post hoc test). (J) Representative electron micrographs (left panels; scale bars, 10 μm) of tibial nerve cross sections from *Lep*-ctrl and *Lep*-AcKO mice, healthy and 2 and 4 wpc. Quantifications (right) of the number of amyelinated and myelinated axons, macrophages, and Schwann cells that contain degeneration profiles was performed and normalized to cross sectional area (21.536 μm², n = 4–5 per group, one-way ANOVA with Tukey’s post hoc test). (K) Analysis of myelin sheath thickness by calculation of the *g-*ratio in data from (J). Scatterplots (right) depict *g*-ratio data points of individual fibers plotted against the respective axon diameter (n = 3 per group, Student’s t test). (L) Representative respirometric traces of the oxygen consumption rate (OCR) in sciatic nerve endoneuria from healthy and injured (4 wpc) sites of control and *Lep*-AcKO mice at 4 wpc. Traces represent the oxygen flux per sciatic nerve mass in mg (left). Quantification shown in the right panel for maximal electron transfer system (ETS_max_) (n = 5 per group, one-way ANOVA and Holm-Šidák multiple-comparisons; M, malate; P, pyruvate; G, glutamate; S, succinate; Cyt*c*, cytochrome *c*; U, chemical uncoupler/CCCP, carbonyl cyanide m-chlorophenyl hydrazone; Rot, rotenone; Ama, antimycin A).

Although the expression of the leptin receptor ligand leptin was not induced in Schwann cells after nerve injury (Figure 2D), *in situ* hybridization revealed sparse leptin mRNA expression in Schwann cells after injury (Figure 6A). Thus, to investigate if injured Schwann cells may undergo leptin autostimulation, we generated *Lep*^*fl/fl*^*::Dhh-Cre* mutant mice that lack leptin expression in Schwann cells (Figure S4D). Leptin ablation from Schwann cells, however, had no impact on nerve regeneration as assessed by electrophysiology (Figure S4E) and histological quantification of remyelinated fibers at 4 wpc (Figure S4F).

The main source of circulating leptin in the body are adipocytes.^24^ In line, we confirmed leptin expression in epineurial adipose tissue by *in situ* hybridization (Figure 6A). Moreover, we observed an increased abundance of leptin protein in paraneural white adipose tissue (WAT) during the time course of peripheral nerve regeneration by ELISA (Figure 6B). Leptin levels have been shown to tightly correlate with adipose tissue mass,^25^ suggesting that adipose tissue may locally respond to peripheral nerve injury. To address this hypothesis, we analyzed the paraneural popliteal adipose tissue and found an increased mass at the injured ipsilateral site during peripheral nerve repair (Figure 6C). This suggests that nerve injury induces an adipocyte response, which supplies Schwann cells with leptin and modulates Schwann cell metabolism via glial leptin receptors, ultimately supporting peripheral nerve repair. In this case, ablation of leptin from adipocytes should recapitulate the phenotype of glial *Lepr*-cKO mutants. Thus, we generated adipocyte-specific leptin knockout mice using an adiponectin promotor-driven inducible CreERT2 line (Figures 6D–6F). We confirmed adipocyte-specificity of tamoxifen-induced recombination by crossing to a TdTomato-flox reporter line (Figure 6D). Importantly, even 4 weeks after nerve injury, we found TdTomato signal to be restricted to epineurial adipocytes in longitudinal nerve sections (Figure 6D). We hence generated *Lep*^*fl/fl*^*::AdiCreERT2* (termed *Lep*-AcKO) mutant mice and treated these mice at the age of 2 months for 3 days with tamoxifen (Figure 6E). After a latency phase of another 2 weeks (to allow depletion of remaining leptin), mutant mice were subjected to sciatic nerve crush injury (Figure 6E). Four weeks after nerve crush, paraneural adipose tissue leptin transcription, as well as endoneural leptin protein concentrations, were significantly reduced in *Lep*-AcKO mice (Figures 6F and 6G). Although ablation of leptin from all adipocytes may entail global metabolic changes, mutant mice displayed only a subtle weight gain (Figure S5A), and no systemic glucose intolerance in the short term of 6 weeks after recombination could be observed (Figure S5B). Notably, digital gait analysis and electrophysiological examination of *Lep*-AcKO mice after nerve crush recapitulated an impaired functional recovery, similar to the findings in glial *Lepr*-cKO mice post nerve injury (Figures 6H and 6I compared with Figures 3C and 3D). Likewise, histological quantification identified impaired axonal regeneration (Figures 6J and S5C), no alteration in Schwann cell numbers and proliferation (Figure S5D), but an increased number of Schwann cells with myelin ovoids in *Lep*-AcKO mice at 2 wpc (Figure 6J), along with a decreased autophagic response as determined by western blot analysis (Figure S5E). Furthermore, *Lep*-AcKO mice recapitulated the impaired remyelination observed in glial *Lepr*-cKO mice at 4 wpc, as reflected by a lower number of remyelinated axons and thinner myelin sheaths (Figures 6J and 6K).

Finally, endoneurial tissue of *Lep*-AcKO mice also demonstrated a significantly impaired gain in the mitochondrial respiratory capacity after injury, as measured by OxPhos protein expression and *ex vivo* respirometry (Figures 6L and S5F). These changes are associated with a decreased OxPhos protein abundance in Schwann cells of *Lep*-AcKO mice at 4 wpc (Figure S5G). Together, adipocyte *Lep*-AcKO mutant mice phenocopy glial *Lepr*-cKO mice on the electrophysiological, histological, and metabolic level after nerve injury, demonstrating that the level of leptin, released from adipocytes, is rate limiting for peripheral nerve regeneration.

### Leptin treatment as a therapeutic rationale to promote regeneration of acutely injured nerves

**Figure 7:**
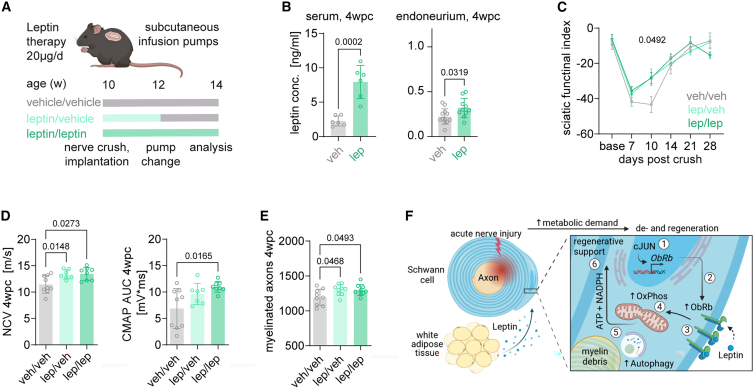
Leptin therapy supports regeneration after peripheral nerve injury (A) Therapeutic leptin treatment regime scheme after acute nerve injury. (B) ELISA for leptin in blood serum (left) and endoneuria (right) of mice receiving continuous leptin treatment (lep/lep) compared with control treatment (veh/veh) at 4 wpc (n = 6–12, Student’s t test). (C) DigiGait walking analysis and calculation of the sciatic nerve functional index (SFI) at different time points post sciatic nerve crush. Baseline was recorded 2 days before sciatic nerve crush (n = 9–10 per group, two-way ANOVA). (D) Electrophysiological measurement of the nerve conduction velocities (NCVs, left) and compound muscle action potentials (CMAPs, right) from ipsilateral sites at 4 wpc (n = 9–10 per group, one-way ANOVA with Tukey’s post hoc test). (E) Histological quantification of the number of remyelinated fibers per tibial nerve cross sections on the semi-thin level at 4 wpc (left; n = 9–10 per group, one-way ANOVA with Tukey’s post hoc test). (F) Schematic model of adipo-glial-mediated catabolism after nerve injury. Schwann cells respond to nerve injury with cJUN-dependent leptin receptor expression (1) and membrane presentation (2). Integration of circulating adipocyte-derived leptin (3) mediates oxidative phosphorylation (4) and myelinophagy (5) to support nerve repair (6).

Finally, we asked whether the identified adipo-glial leptin signaling axis can be targeted to improve nerve repair. To this end, we performed an exploratory three-armed treatment trial during which mice received subcutaneous leptin (lep) or vehicle (veh) infusion pumps for the first 2 weeks after nerve crush. The infusion pumps where then replaced once and kept for another 2 weeks, resulting in three groups: (1) veh/veh, (2) lep/veh, and (3) lep/lep (Figure 7A). Notably, leptin treatment increased the level of circulating and endoneural leptin when measured at 4 wpc (Figure 7B). When we next assessed functional nerve regeneration by digital gait analyses, we found an improved sciatic nerve functional index (SFI) in both groups that received leptin in the first 2 weeks after crush (Figure 7C). In addition, electrophysiological examination of injured sciatic nerves at the end of the study confirmed an improved nerve repair upon leptin treatment with regard to the NCV and CMAPs (Figure 7D). In line, histological analysis of nerve regeneration confirmed a positive therapeutic effect, with an increased number of remyelinated axons 4 weeks after injury in the two leptin treatment groups (Figure 7E). Taken together, these data demonstrate, in proof of principle in an experimental nerve crush model, that leptin treatment is effective to support peripheral nerve regeneration after acute nerve injury.

## Discussion

Peripheral nerve regeneration is characterized by a dramatic remodeling of the nerve distal to the injury site. Repair Schwann cells are the key cellular players that ensure and convey the associated de- and regenerative events in the injured nerve, including the breakdown of myelin debris, the facilitation of axonal regrowth and eventually, the remyelination of newly regenerated axons. However, how repair Schwann cells adapt metabolically to complete this complex sequence of cellular events remains ill-defined. We demonstrate here that peripheral nerve regeneration induces a profound metabolic activation of repair Schwann cells, including an oxidative switch and a marked increase in mitochondrial respiration.

Using conditional mutagenesis approaches in mice, we identified the leptin/leptin receptor signaling axis as an upstream regulatory mechanism of the injury-associated metabolic repair response in Schwann cells, which ensures efficient peripheral nerve repair (Figure 7F). We thereby demonstrate, in proof of principle, the fundamental relevance of dynamic metabolic adaptation by repair Schwann cells in the course of nerve regeneration.

Recently, Schwann cells have been shown to respond with glycolytic activation immediately (within 72 h) after acute nerve injury, a period that covers the onset of Wallerian degeneration.^12^ We show here that the subsequent time course of nerve de- and regeneration (7–28 days post crush) is associated with an induction of OxPhos in repair Schwann cells. In general, nerve de- and regeneration are characterized by catabolic autophagy and anabolic remyelination in repair Schwan cells as early and late events, respectively. Notably, we demonstrate that leptin receptor expression is induced in Schwann cells already at 3 days post crush and affects both myelin autophagy (1–2 wpc) and remyelination (2–4 wpc) and furthermore induces mitochondrial expansion and respiration throughout peripheral nerve regeneration (2–4 wpc). Indeed, repair Schwann cell catabolism and anabolism are likely to represent continuous, closely interdependent processes, where efficient anabolic remyelination can only occur after efficient catabolic myelin debris removal, and our data suggest that mitochondrial OxPhos acts as a functional link between both tasks. In line with recent findings in cultured cancer cells,^26^ we demonstrate that autophagy and mitochondrial respiration are metabolically coupled in repair Schwann cells. It is therefore tempting to hypothesize that former myelin lipids may be recycled, at least in part, to serve as energy substrate for mitochondria during remyelination. In agreement with this model, the stimulating effect of leptin on mitochondrial respiration was blocked when autophagy or fatty acid oxidation were inhibited. Leptin signaling may hence divert myelin derived fatty acids to fuel mitochondria, energy production, and ultimately remyelination.

Notably, the consequences of glial leptin receptor ablation for nerve repair exceed those of mouse mutations that specifically affect Schwann cell autophagy, such as in conditional glial *Atg7* or calcineurin mutants, which display only a minor delay of nerve regeneration with normal remyelination.^5^^,^^22^^,^^27^ Glial leptin receptor mutants, however, also show a transient impairment of Schwann cell proliferation after injury, an energy-demanding process, which was not impaired in Schwann cell autophagy mutants.^5^^,^^22^^,^^27^ These differences could result from compensatory mechanisms and signaling pathways in the aforementioned autophagy mouse mutants and/or by a more general role of leptin receptor signaling in the regulation of Schwann cell catabolism and autophagy. An additional, autophagy-independent modulation of mitochondrial expansion and metabolism is suggested by blunted mitochondria-related gene transcription and mitochondrial morphological changes in leptin receptor mutant mice. However, these changes may also result from a reduced energetic demand upon leptin receptor ablation and hence constitute a secondary phenomenon. In general, the impact of disrupted leptin receptor signaling on nerve regeneration is moderate and comparable to mouse mutants where mTOR, a central metabolic hub, is ablated from Schwann cells,^28^ indicating the existence of compensatory mechanisms. Indeed, the interplay of metabolic push (substrate supply) and pull (energetic demand) between autophagy and OxPhos is still poorly understood^29^ and also ill-defined in Schwann cells. Hence, to which extent metabolic flexibility is determined by substrate supply or by energetic demand in repair Schwann cells remains an important question to be addressed in the future.

We show that the activation of leptin receptor signaling in repair Schwann cells involves the expression and translocation of receptors to the plasma membrane and results in glial metabolic modulation during peripheral nerve repair. Leptin, next to possible other stimuli, thereby activates the JAK/STAT3 pathway. Importantly, the adipose tissue origin of leptin highlights a novel non-cell-autonomous aspect to nerve repair and adds a new layer of understanding to how Schwann cell repair responses are modulated. Indeed, we show that adipocytes respond to peripheral nerve injury with an increase in the mass of paraneural adipose tissue ipsilateral to the injury site, which is associated with a respective increase in local leptin levels after nerve injury. Although identification of the detailed mechanisms that lead to paraneural white adipose tissue increase after nerve injury are beyond the scope of the present study, adipocyte expansion has also been shown in wounded skin and may constitute a general injury response.^30^ In addition, adipose tissue-derived stem cells are well known for their regenerative capacity including in injured nerves.^31^^,^^32^ We show in proof of principle that adipocyte-derived leptin positively promotes peripheral nerve repair by Schwann cells. In fact, the redundant consequences of conditional ablation of leptin from adipocytes and of the leptin receptor from Schwann cells on the functional, histological, and molecular level demonstrate a novel adipo-glial signaling axis in nerve repair. Although a local paraneural adipose tissue response may suggest a local route of epineurial leptin to endoneural leptin receptor signaling in diseased nerves, circulating leptin may additionally stimulate glial leptin receptors. This concept is supported by the positive therapeutic outcome in injured mice that were treated with subcutaneous leptin infusion pumps. Indeed, in the investigated crush model, therapeutic administration of recombinant leptin resulted in improved nerve regeneration.

However, the regenerative potency of leptin may be different in other, more severe types of nerve injury, as suggested by lower treatment efficiency after experimental nerve transection, and requires careful consideration of potential side effects due to the expression of the leptin receptor in various cell types.^33^^,^^34^ Nevertheless, the modulation of Schwann cell energy metabolism and peripheral nerve repair by the leptin/leptin receptor signaling module could represent a novel, translatable treatment strategy where regeneration is poor.^35^ Moreover, the so far neglected dimension of adipocyte-glial interaction in nerve repair may harbor implications for other, chronic nerve injuries, especially of those with a metabolic component such as diabetic neuropathy.

### Limitations of the study

The full relevance of our findings that leptin receptor signaling increases autophagy and mitochondrial respiration in Schwann cells after acute nerve injury requires further studies. Although the specific impact of autophagy to nerve repair has been addressed by several studies using conditional mouse mutants, no such study has been published yet for specific disruption of mitochondrial respiration in Schwann cells after nerve injury. Thus, it remains to be established to what extent energy production by mitochondria in Schwann cells contributes to nerve repair. Moreover, elaboration of the precise nature of the adipose tissue response to nerve injury (next to adipogenesis and leptin production) requires more research and may lead to the discovery of further pro-regenerative mechanisms in the future. In this study, mice from both male and female sexes have been used, equally distributed between groups. However, sample sizes are too low to allow interpretation of the data for the individual sex.

## STAR★Methods

### Key resources table

**Table:** 

REAGENT or RESOURCE	SOURCE	IDENTIFIER
**Antibodies**		

Rabbit Anti-Dendra2	Antibodies-online.com	cat.#:ABIN361314;RRID: AB_10789591
Rabbit Anti-Leptin/Obese Receptor (OBRb)	Bio Trend	Cat#. OBR12-A; RRID: AB_1611942
Rabbit Anti-S100beta	Abcam	cat.#: ab52642; RRID: AB_882426
Chicken Anti-MBP	Invitrogen	cat.#: PA1-10008; RRID: AB_1077024
Rabbit Anti-Iba1	Wako	cat.#: 019-19741; RRID: AB_839504
Rabbit Anti-Sox10	Abcam	Cat.#: ab180862; RRID: AB_2721184
Mouse Anti-PCNA	Abcam	Cat.#:ab29; RRID: AB_303394
Mouse Anti-Total OXPHOS cocktail	Abcam	cat.#: ab110413; RRID: AB_2629281
Chicken Anti-Neurofilament H	Bioloegend	Cat.# 822601; RRID: AB_2564859
Goat Anti-PDGFRa	R&D systems	Cat.# AF1062; RRID: AB_2236897
Rabbit Anti-LC3B	Cell signaling	cat.#: 2775; RRID: AB_915950
Rabbit Anti-p62	Cell Signaling	cat.#: 5114S; RRID: AB_10624872
Rabbit Anti-pERK	Cell Signaling	cat.#: 9101S; RRID: AB_331646
Rabbit Anti-ERK	Cell Signaling	cat.#: 4695; RRID: AB_390779
Rabbit Anti-HK I	Cell Signaling	cat.#: 2024S; RRID: AB_2116996
Rabbit Anti-HK II	Cell Signaling	cat.#: 2867S; RRID: AB_2232946
Rabbit Anti-pAKT	Cell Signaling	cat.#: 4060; RRID: AB_2315049
Rabbit Anti-AKT	Cell Signaling	cat.#: 4691; RRID: AB_2617178
Rabbit Anti-pSTAT3	Cell Signaling	Cat.#: 9145; RRID: AB_2491009
Rabbit Anti-STAT3	Cell signaling	Cat.#: 12640; RRID: AB_2629499
HRP-goat-anti-rabbit	Cell Signaling,	cat.#: 7074; RRID: AB_2099233
HRP-horse-anti-mouse	Cell Signaling	cat.#: 7076; RRID: AB_330924
Cy3-goat-anti-rabbit	Jackson Immuno Research	cat.#:111-165-144; RRID: AB_2338006
Cy3-goat-anti-mouse	Jackson Immuno Research	cat.#:115-165-071; RRID: AB_2338687
Alexa Fluor Plus 488 Goat anti-Chicken IgY (H+L)	Invitrogen	cat.#: A32931; RRID: AB_2762843
Alexa Fluor 488 Goat Anti-Rabbit	Jackson Immuno Research	cat.#: 111-545-144; RRID: AB_2762843
Alexa Fluor 555 Donkey Anti-Rabbit	Invitrogen	Cat.# A31572; RRID: AB_162543
Alexa Fluor 488 Donkey Anti-Goat	Invitrogen	Cat.#: A32814; RRID: AB_2762838
Cy5-Donkey Anti-Mouse	Jackson Immuno Research	Cat.#: 715-175-151; RRID: AB_2340820
AffiniPure Fab Fragment Donkey Anti-Mouse IgG (H+L)	Jackson Immuno Research	Cat.#: 715-007-003; RRID: AB_2307338
Alexa Fluor 488 Goat Anti-Rabbit	Jackson Immuno Research	cat.#: 111-545-144; RRID: AB_2338052
Wheat Germ Agglutinin-Alexa Fluor 488 Conjugate	Invitrogen	cat.#: W11261

**Chemicals, peptides, and recombinant proteins**		

Recombinant Leptin	R&D Systems	Cat.#: 498-OB
3 Methyl Alanine (3MA)	MedChemExpress	Cat.#: HY-19312
Etomoxir	MedChemExpress	cat.#: HY-50202
C188-9 (Stat3 Inhibitor)	MedChemExpress	cat# HY-112288
Ammonium Chloride (Lysosomal Inhibitor)	Sigma	Cat# A4514
Proteinase K	Sigma	Cat# 2308
Tamoxifen	Sigma	Cat.# T5648
EGTA	Sigma	cat.#: E4378
Magnesium Chloride	Scharlau	cat.#: MA 0036
K-lactobionate	Sigma	cat.#: 153516
Malate	Sigma	Cat.# M1000
Pyruvate	Sigma	Cat.# P2256
Glutamate	Sigma	Cat.# G1626
Adenosine Di-Phosphate	Calbiochem	Cat.# 117105
Cytochrome C	Sigma	Cat.# C7752
Succinate	Sigma	Cat.# S2378
CCCP	Sigma	Cat.# C2759
Rotenone	Sigma	Cat.# R8875
Antimycin A	Sigma	Cat.# A8674
Oligomycin	Sigma	Cat.# O4876
FCCP	Sigma	Cat.# C2920
Glutaraldehyde	Electron Microscopy Sciences	Cat.# 16220
Paraformaldehyde	Electron Microscopy Sciences	Cat.# 15710
Taurine	Sigma	cat.#: T0625
Potassium Dihydrogen Phosphate	Merck	cat.#: 104873
HEPES	Sigma	cat.#: H7523
cOmpleteTM Protease Inhibitor Cocktail (Roche)	Sigma	Cat.#: 4693132001
PhosSTOP (Roche)	Sigma	Cat.#: 4906837001
Fast Green FCF	Sigma	Cat.#: F7252
D-Sucrose	Roth	cat.#: 4621.1
Bovine Serum Albumin	Sigma	cat.#: A6003

**Critical commercial assays**		

Quant-iT PicoGreen dsDNA Assay	Thermo Fisher Scientific	Cat.#: P7589
BaseScope Detection Reagents v2– RED	ACD Bio	Cat.#: 323910
Phospho Explorer Antibody Microarray	Fullmoonbio	Cat.#: PEX100

**Deposited data**		

Accession Number for bulk RNA Seq data	N/A	GEO: GSE244328
Unprocessed data underlying display items (source data)	N/A	Data S1

**Experimental models: Organisms/strains**		

LepR-Flox: B6.129P2-Leprtm1Rck/J	The Jackson Laboratory	RRID: IMSR_JAX:008327
Dhh-Cre: FVB(Cg)-Tg(Dhh-cre)1Mejr/J	Dies Meijer Lab	RRID: IMSR_JAX:012929
PhAM Dendra2: B6;129S-Gt(ROSA)26Sortm1(CAG-COX8A/Dendra2)Dcc/J	The Jackson Laboratory	RRID: IMSR_JAX:018385
db/db: BKS.Cg-Dock7m +/+ Leprdb/J	The Jackson Laboratory	RRID: IMSR_JAX:000642
Lep-Flox: Leptm1.1Gvcao	Odle et al.^36^	RRID: MGI:5881975
cJUN –Flox: Juntm1Bdc	Behrens et al.^37^	RRID: MGI:3703004
TdTomato: B6.Cg-Gt(ROSA)26Sortm14(CAG-tdTomato)Hze/J	The Jackson Laboratory	RRID: IMSR_JAX:007914
Mpz-Cre: B6N.FVB-Tg(Mpz-cre)26Mes/J	The Jackson Laboratory	RRID: IMSR_JAX:017927
Adi-CRE ERT2: C57BL/6-Tg(Adipoq-icre/ERT2)1Soff/J	The Jackson Laboratory	RRID: IMSR_JAX:025124

**Oligonucleotides**		

qPCR Primers	This study	Refer to Table under “RNA preparation, quantitative real-time PCR (qPCR) analysis” section.
Base Scope Mm-Lepr-02 Probe	ACD Bio	cat.#: 804681, mRNA accession NM_146146.3, target region: 2721–3610
Base Scope Mm-Lep Probe	ACD Bio	cat#: 513671, mRNA accession NM_008493.3, target region: 2–884

**Software and algorithms**		

Evidence 3102 software (Neurosoft Version 3.7.3.7)	Schreiber and Tholen Medizintechnik	N/A
DatLab software (version 7)	OROBORUS Instruments	N/A
STAR Alignment Tool (version 2.5)	Dobin et al.^38^	N/A
featureCounts Tool	Liao et al.^39^	N/A
BiomaRt (v2.46.3)	Durinck et al.^40^	N/A
DESeq2 (v1.31.5)	Love et al.^41^	N/A
R/bioconductor environment (version 4.0.5)	http://new.bioconductor.org/	N/A
Seahorse Wave Desktop (version 2.6.3)	Agilent Technologies	N/A
MARS Data Analysis software (version 3.32	BMG Labtech	N/A
Fiji (v 1.53)	Imagej.net	N/A
Ingenuity Pathway Analysis (IPA)	Qiagen	N/A
qPCRSoft384 Version 1.1	Analytik Jena	N/A

**Other**		

Alzet Micro-Osmotic Pump	Alzet	Cat.# 1002
DigiGait system	Mouse Specifics, Inc	N/A

### Resource availability

#### Lead contact

Further information and requests for resources and reagents should be directed to and will be fulfilled by the lead contact, Robert Fledrich (Robert.Fledrich@medizin.uni-leipzig.de).

#### Materials availability

This study did not generate new unique reagents.

#### Data and code availability

Bulk RNA sequencing data have been deposited at GEO and are publicly available as of the date of publication. The accession number is GSE244328. This paper does not report original code. The source data which has been used to generate the data panels in the figures can be found in Data S1 – Source Data. Any additional information required to reanalyze the data reported in this paper is available from the lead contact upon request.

### Experimental model and study participant details

#### Animal models

All mice used for this study include *Lepr-flox* (RRID: IMSR_JAX:008327),^20^ *Dhh*-Cre driver (RRID: IMSR_JAX:012929),^42^ PhAM Dendra2 (RRID: IMSR_JAX:018385); *Lep-flox* (RRID: MGI:5881975)^36^; *TdTomato* (RRID: IMSR_JAX:007914)^43^; *Adi-CreERT2* (RRID: IMSR_JAX:025124),^44^ *db/db* (RRID: IMSR_JAX:000642),^45^ *cJun*-flox (RRID: MGI:3703004),^37^ and *Mpz*-Cre (RRID: IMSR_JAX:017927).^46^ All mice were on a C57BL6 background. Animal experiments were approved by the Landesdirektion in Sachsen and conducted according to the respective regulations for animal experimentation. Criteria for experimental inclusion and exclusion were defined a priori. Experimental groups were randomly determined by the genotype, age and weight. Animal health was regularly monitored. During or after the experiment, animals were excluded when an impaired health condition, not attributed to the genotype or experiment, was observed according to the veterinary or a weight loss of >10% of the average group occurred. No animals had to be excluded from any of the experiments due to weight loss or ailment. The Grubbs’ test (= ESD method) was used for determination of exclusion criteria concerning the outcome assessment. Statistical analysis was performed using Graph Pad (Prism) software.

Both sexes, male and female, have been used equally distributed between the groups. Only mice between two and four months of age (age-matched for each experiment) have been used. Wherever possible, littermate controls have been used. Mice were kept on a standard diet (D12450B, Research Diets, New Brunswick), in individually ventilated cages at 22 +/-2°C temperature, with a light/dark cycle of 12h/12h per day. The health status of the mice was checked on a daily basis. The investigator was blinded towards the genotype of the animals during all animal experiments encompassing phenotype analyses, electrophysiology, nerve crush, and histology. Samples from experimental animals for all molecular and histological analyses were selected randomly from the experimental groups in a blinded fashion.

#### *Ex vivo* sciatic nerve culture

Sciatic nerves of adult mice were dissected and the epineurium/perineurium was desheathed using fine forceps. The explants were then directly transferred into serum-free ex vivo medium (DMEM:F12 containing 17.5mM Glucose, 0.5mM Pyruvate, 1X Glutamax, 1X Antibiotic/Antimycotic and 1X N2 supplement). The nerve pieces were then incubated in 1 ml ex vivo medium supplemented with either 1 μg/ml leptin (R&D, cat.#: 498-OB) dissolved in the media or 50 mM 3-methyladenine (3MA, MedChemExpress, cat.#: HY-19312 ) dissolved in the media or 50 μM Etomoxir (MedChemExpress, cat.#: HY-50202 ) dissolved in ddH2O or 1uM C188-9 (MedChemExpress, cat# HY-112288) dissolved in DMSO or 15mM NH_4_Cl (Sigma Cat# A4514) dissolved in ddH_2_O. The nerves were incubated for 6 days *in vitro* at 37°C and 5% CO2. The medium was changed every 3 days. After incubation, the samples were removed from cell culture media and immediately used and lysed for protein analysis, histology or Seahorse assays.

### Method details

#### Genotyping

Genotyping was performed via PCR amplification of genomic DNA. The DNA was isolated from tail biopsies via incubation in 1X Modified Gitschier Buffer containing 0.5% TritonX-100 and 1 mg/ml proteinase K for at least 2 h at 55°C under constant shaking. Proteinase K was subsequently heat inactivated at 90°C for 10 min. PCR primers were employed in a co-amplification reaction.

#### Tamoxifen injections

Adipocyte reporter (*TdTo*^*fl/fl*^*::AdiCreERT2*) and conditional adipocyte leptin knockout (*Lep*^*fl/fl*^*::AdiCreERT2*) mice received i.p. injections with 30 mg/kg tamoxifen dissolved in ethanol and corn oil at the age of 8 weeks for three consecutive days. Sciatic nerves were then crushed when mice reached the age of 10 weeks. The crush was performed as described in the “sciatic nerve crush” section. Pain management was conducted by adding Metamizole (500 μl per 100 ml drinking water) from 1 day before the crush until 3 days post crush. Animals were analyzed at 28 days post crush. Blood and tissue samples were collected afterwards. Blood was immediately collected by heart puncture with a syringe after mice were euthanized by cervical dislocation.

#### Leptin therapy

Osmotic Alzet pumps (Alzet Model# 1002, pump rate 0.25 μl/h for 14 days) were prepared according to the manufactures instructions in a laminar flow hood at room temperature. All used solutions were sterilized beforehand. Briefly, recombinant leptin (R&D, cat.#: 498-OB) was dissolved in 0.9% sterile NaCl solution at 3.3 mg/ml. With a volume of 100 μl and a flow rate of 0.25 μl/h, treated animals received a total of 20 μg leptin per day. The pumps were filled with either 100 μl of leptin solution (leptin treatment) or 0.9% sterile NaCl solution (vehicle treatment) after which flow moderators were inserted. The filled pumps were primed for 6 h in sterile 0.9% NaCl solution at 37°C. Animals designated for the leptin treatment experiment were anesthetized at the indicated timepoints with a combination of 0.5 mg/kg Medetomidin (Domitor), 5 mg/kg Midazolam (Dormicum) and 0.05 mg/kg Fentanyl, and a nerve crush was performed as described in the “sciatic nerve crush” section. After suturing the incisure at the sciatic nerve, a second incisure was generated at the neck of the animals. The pump was implanted subcutaneously and the incisure sutured subsequently. Animals were monitored closely until they woke up completely and were able to eat and drink properly. Pain management was conducted by adding Metamizole (500 μl per 100 ml drinking water) from 1 day before the crush until 3 days post crush. The pump of each mouse was replaced after two weeks. Metamizole was also added to the drinking water 1 day before until 3 days after pump replacement surgery. Animals in the vehicle/vehicle and leptin/leptin groups each received a new pump pretreated as the first one. The second pump implanted in the animals from the leptin/vehicle group was filled with sterile 0.9% NaCl solution. Gait analysis was performed at the indicated timepoints until 4 weeks post crush. At the last time point, electrophysiological analysis was performed and blood and tissue samples collected. Blood was immediately collected by heart puncture in deep anesthetized mice with a syringe right before euthanization by cervical dislocation.

#### Sciatic nerve crush

Animals were anesthetized at the indicated timepoints with a combination of 0.5 mg/kg Medetomidin (Domitor), 5 mg/kg Midazolam (Dormicum) and 0.05 mg/kg Fentanyl and placed on the belly. The hindlimbs were gently spread and fixed with tape. After exposure of the sciatic nerve by a small incisure, the nerve was crushed according to a standardized protocol concerning pressure, localization and duration of compression using artery forceps. The nerve was compressed at the mid-femoral level for 40s. The incisure was sutured and animals were monitored closely until they woke up completely and were able to eat and drink properly. Pain management was conducted by adding Metamizole (500 μl per 100 ml drinking water) from 1 day before the crush until 3 days post crush. The mice were sacrificed after the indicated time periods. Ipsilateral nerves were dissected at the crush site and either directly immersed in Karlsson and Schultz fixation solution (4% PFA and 2.5 glutaraldehyde in 0.1 M phosphate buffer) for light microscopic and electron microscopic analysis or in 4% PFA solution for immunohistochemical staining. Contralateral nerves were removed completely and processed identical to the ipsilateral side. For molecular analyses, ipsi- and contralateral nerves were separately placed in ice cold PBS after dissection. For molecular biological analyses, the perineurium was thoroughly removed from each nerve. Peri- and endoneurium were then separately shock frozen with dry ice until further processing. Additionally, the paraneural popliteal white adipose tissue depot was collected from each side, lymph nodes removed and separately shock frozen on dry ice. Control mice were handled identical to crushed animals. After anesthesia, the sciatic nerve was exposed and the incisure closed without conducting nerve compression. In cases of molecular analyses of crushed and control sciatic nerves, only the endoneurial compartment of nerve tissue was prepared always with the perineuria stripped off (unless indicated otherwise).

#### DigiGait walking analysis

Gait analysis was performed in a fully automated and standardized manner using the DigiGait system (Mouse Specifics). Mice were placed on a treadmill moving at a constant speed of 20 cm/s. The gait of the animals was recorded through the transparent treadmill by a video camera placed below. Animals were trained for 3 consecutive days before recording of the baseline to ensure that animals were familiar with the environment and able to constantly run for at least 6 seconds on the treadmill. After a one day break, animals received surgery and were then analyzed at the indicated timepoints. The speed of the treadmill was increased incrementally while recording started at the top speed of 20 cm/s. Every mouse was recorded for at least 5 seconds of constant walking without distraction. The video material was automatically evaluated using the DigiGait analysis software (Mouse Specifics) to calculate the sciatic functional index.^47^

#### Electrophysiology

For electrophysiological examination mice were anesthetized with a combination of 0.5 mg/kg Medetomidin (Domitor), 5 mg/kg Midazolam (Dormicum) and 0.05 mg/kg Fentanyl and placed on their belly under an infrared lamp to maintain a constant body temperature. Pairs of stainless-steel needle electrodes (Schreiber & Tholen Medizintechnik) were placed subcutaneously along the nerve at the sciatic notch for proximal stimulation and along the tibial nerve above the ankle for distal stimulation. Recording electrodes were placed in the lower foot muscle and between the second and third walking pad. The grounding electrode was placed near the chest below the right forelimb. Both, proximal and distal stimulation were conducted with square wave pulses with 3 mM lasting 0.1 ms using the Evidence 3102 electroneurography device (Schreiber & Tholen Medizintechnik). The nerve conduction velocity (NCV) was determined by the latencies between proximal and distal recordings and the distance of the stimulation electrodes. The distance was measured alongside the skin surface while the legs of the mice were fully extended. The compound muscle action potential (CMAP) was assessed as the area under the curve (AUC) of the recordings and automatically calculated by the Evidence 3102 software (Neurosoft Version 3.7.3.7, Schreiber & Tholen Medizintechnik).

#### Oroboros *ex vivo* high resolution respirometry

Mitochondrial respiration *ex vivo* analysis was performed in mouse sciatic nerve tissue using the high-resolution Oxygraph-2k (OROBOROS Instruments). Briefly, whole left and right sciatic nerves were dissected, the perineurium was immediately removed and endoneuria fluffed using a pair of fine forceps and placed in mitochondrial respiration medium Mir05 (0.5 mM EGTA (Sigma Aldrich, cat.#: E4378), 3 mM MgCl_2_·6H_2_O (Scharlau, cat.#: MA 0036), 60 mM K-lactobionate (Sigma Aldrich, cat.#: 153516), 20mM Taurine (Sigma Aldrich, cat.#: T0625), 10 mM KH_2_PO_4_ (Merck, cat.#: 104873), 20 mM HEPES (Sigma Aldrich, cat.#: H7523), 110 mM D-Sucrose (Roth, cat.#: 4621.1), 1g/l fatty acid free BSA (Sigma Aldrich, cat.#: A6003)). Experiments were carried out under atmospheric oxygen conditions (∼200 nM/mL O_2_) performing a multiple substrate-uncoupler-inhibitor titration (SUIT) protocol at 37°C with following substrate concentrations: 0.5mM Malate (Sigma Aldrich, cat.#: M1000) + 5mM Pyruvate (Sigma Aldrich, cat.#: P2256) + 10mM glutamate (MPG, (Sigma Aldrich, cat.#: G1626)), 5mM ADP (complex I-coupled respiration, Calbiochem, cat.#: 117105), 10μM cytochrome c (integrity of outer mt-membrane, (Sigma Aldrich, cat.#: C7752)), 10mM succinate (complex I&II-coupled respiration, (Sigma Aldrich, cat.#: S2378)), stepwise 0.5μM CCCP (ETS_max_, maximal electron transfer system, Sigma Aldrich, cat.#:C2759), 0.5μM rotenone (Sigma Aldrich, cat.#: R8875) + 2.5μM antimycin A (residual oxygen consumption, ROX, data not shown, Sigma Aldrich, cat.#: A8674). Oxygen flux was quantified using DatLab software (version 7, OROBOROS Instruments). Oxygen consumption rate (OCR) was normalized to ROX levels and nerve fiber wet weight of dry blotted fiber bundles. The investigators performing the respiratory analyses were blinded to the sample group allocation during the experiment and analysis of the experimental outcome.

#### Seahorse Mitostress Assays

Sciatic nerves from mice were dissected and cultured ex vivo with different compounds as indicated in the ‘Ex vivo sciatic nerves cultures’ section for a period of 6 days. Each nerve fragment was then transferred into a single well of a Seahorse XF Islet Capture Plate containing 500uL of Seahorse XF DMEM media supplemented with 17.5mM Glucouse, 0.5mM Pyruvate and 2mM Glutamine. Upon transfer, the nerves were washed twice with the media and then incubated for 1 hour at 37°C in a non-CO2 incubator before the Mitostress Assay. The assay was designed to measure 4 cycles of basal respiration, 5 cycles after Oligomycin injection (Sigma O4876, 20uM Final concentration), 5 cycles after FCCP injection (Sigma C2920, 20uM Final Concentration) and 5 Cycles after Rotenone+AntimycinA injection (Sigma R8875, Sigma A8674, 20uM final concentration of both compounds). Upon completion, total DNA was extracted from the nerve fragments by homogenization in DNA extraction buffer (100mM Tris-HCl pH 8, 10mM EDTA, 200mM NaCl) followed by ProteinaseK digestion (1mg/mL) at 55°C overnight. The following day, proteinaseK was heat inactivated at 95°C for 10 mins and total DNA was then measured using the Quant-iT PicoGreen dsDNA Assay (P7589, Thermo) according to the prescribed protocol. Oxgen Consumption Rates were then normalized to 1ug of total DNA for each well. A total of 4 – 5 wells (n = 4-5 animals) were used for each experimental condition. OCR and ECAR values were calculated using the Wave Desktop Software (version 2.6.3)

#### Light microscopy, electron microscopy and morphometry

Mice were euthanized at the indicated time points, sciatic and tibial nerves were removed and fixed in Karlsson and Schultz solution (4% PFA and 2.5 glutaraldehyde in 0.1 M phosphate buffer) for at least 7 days at room temperature. Afterwards, nerves were fixed and contrasted with osmium tetroxide and uranyl acetate and embedded in Agar 100 epoxy resin. 500 nm thick semi-thin cross sections were generated using a microtome (Powertome X, RMC) and a Histo Diamond knife (45° 8.0 mm, Diatome). Sections were placed on a microscope slide, stained with AzurII/methylene blue for 1 min at 60°C and mounted with Eukitt. Light microscopic images were captured fully automated using a Axio Scan.Z1 (Zeiss) at a 40-fold magnification. For electron microscopic analysis, 50 – 70 nm thick ultra-thin cross section were obtained using the same microtome as above but equipped with an Ultra Diamond knife (45° 2.1 mm, Diatome). Ultra-thin sections were placed on a copper carrier grid and contrasted with 1% uranylacetate and lead citrate.^48^ Electron microscopic images were taken on a Sigma EM electron microscope equipped with a STEM device (Zeiss) at a 3000-fold magnification. Quantification of histopathological parameters on LM images was conducted over the entire cross section, to quantify the number of myelinated axons. For EM analysis, at least 20 random pictures were taken per nerve, and the number of myelinated and amyelinated axons, Schwann cell nuclei, Schwann cells with myelin ovoids and macrophages were quantified per area. Myelin sheath thickness was determined by *g*-ratio analysis by dividing the axonal diameter by the fiber diameter (diameter of axon including the myelin sheath). The diameters of the fibers/axons were calculated by the measured areas (not perimeters), respectively. At least 200 axons were quantified for the *g* ratio per animal. For mitochondria morphometric analysis, EM images were taken with a magnification of 20k-fold. The number of mitochondria per μm^2^ Schwann cell cytoplasm, the mitochondrial size in μm^2^ and the percentual occupancy (mitochondrial area/Schwann cell cytoplasmic area) were quantified. High pressure freezing followed by freeze substitution and resin embedding were performed as published in Möbius et al.^49^ In brief nerves were dissected and immediately transferred to phosphate buffered, 4% paraformaldehyde and 2.5% glutardialdehyde containing fixative^50^ for short immersion fixation while trimming of nerves to protect from physical damage. Tibial nerves were aligned in specimen carrier with 2mm x 0.2mm indentation embedded in polyvinylpyrrolidone (PVP) as cryoprotecting filler and covered by a flat lid. The sandwich was mounted in the holder cartridge for liquid nitrogen based high pressure freezing with the EM HPM100 (Leica Microsystems). Samples were stored in liquid nitrogen. Frozen samples were further processed by automated freeze substitution using an AFS-2 (Leica, Vienna, Austria) to slowly substitute vitrified ice by organic solvents. In brief, samples were infiltrated at -90°C with 0.1% tannic acid in acetone for 100h, washed with chilled acetone followed by fixation and contrasting using 2% OsO4 (EMS) in acetone additionally containing 0.1% uranyl acetate for 7h at -90°C. Subsequently temperature was raised slowly from -90°C to 4°C within the next 33h in the fixative. Following acetone washing steps with concurrent warm up to room temperature. Substituted tissue was further infiltrated with increasing mixtures of Epon resin (Serva) in acetone up to 100% resin infiltration for 31h at RT with several changes of pure Epon. Remaining filler was removed from specimen. Tissue was aligned in resin-containing embedding molds and subsequently polymerized at 60°C.

#### Leptin ELISA

Leptin abundances were determined for sciatic nerve endoneurium, white adipose tissue and blood serum with the Mouse/Rat Leptin Quantikine ELISA Kit (Cat.#: MOB00, R&D) according to the manufacturers instructions. Blood serum was derived from whole blood by clotting for 2 h at room temperature following a centrifugation for 20 min at 2000 g. Blood serum (upper phase) was removed, aliquoted and stored at -20°C until further use. Fresh frozen sciatic nerve endoneurium and white adipose tissue were homogenized in ice cold RIPA buffer (50 mM TRIS pH 8, 150 mM NaCl, 1% v/v Triton X-100 reduced, 0.5% w/v sodium deoxycholate, 0.1% w/v sodium dodecyl sulfate, cOmplete Protease Inhibitor Cocktail (Roche), PhosSTOP Phosphatase Inhibitor Cocktail (Roche)) using the Bead Ruptor 24 (Omni). After a centrifugation for 10 min at 10.000 rpm at 4°C, the soluble phase was removed and protein quantification performed. For endoneuria and blood samples, protein concentrations were determined via tryptophan fluorescence (WF) of unfolded proteins according to Wiśniewski and Gagauz,^51^ with modifications. In brief, samples were diluted in WF buffer (7.6 M Urea, 30 mM Tris, pH 8, 1 % SDS, 50 mM DTT) and heated at 99 °C for 15 min. Fluorescence was measured with a fluorescence spectrometer (295 nm excitation, 341 nm emission) in a microcuvette. Protein concentrations were calculated from fluorescence values of samples and a tryptophan standard, assuming a tryptophan content of 1.17 % in the samples. Blood serum was diluted 20-fold with calibrator diluent RD5-16. Sciatic nerve endoneurium samples were diluted to 1 mg/ml with RIPA buffer before conducting the ELISA, in order to measure leptin concentration relative to endoneurial protein. For the paraneural white adipose tissue, the complete popliteal fat depots were collected, the lymph nodes removed, the tissue lysed as a whole in equal volumes of RIPA buffer respectively, to measure and calculate the total leptin content in the fat depots. The same volume of all samples and standards were pipetted onto the pre-coated ELISA 96-well plate and incubated for 2 h on room temperature under constant shaking. After extensive washing, mouse/rat leptin conjugate was added to each sample following a further incubation for 1 h at room temperature under constant shaking. The plate was again washed extensively and all wells were incubated with streptavidin-HRP 1 solution for 30 min at room temperature under constant shaking. After a last washing, substrate solution was added and incubated for 30 min at room temperature and protected from light. Stop solution was added and the optical density at 450 nm was detected using a wavelength correction measurement at 540 nm in a SPECTROstar nano (BMG Labtech) plate reader. The standard curve equation was calculated by a standard row of recombinant leptin concentrations provided with the kit using MARS Data Analysis software (version 3.32, BMG Labtech) and subsequently used to calculate leptin concentration in the samples. If the leptin concentration of a sample exceeded or was below the standard curve range, ELISA was repeated with an increased or decreased dilution of the sample, respectively.

#### Immunohistochemistry

Sciatic and tibial nerves were dissected from the mice at the indicated time points and fixed in 4% PFA at 4°C overnight. After a maximum of 24 hours, the nerves were embedded into paraffin. 5 μm thick sections were cut from the paraffin blocks and mounted of Superfrost Plus slides (Thermo Scientific). The samples were deparaffinized during a standard xylol and ethanol series. Further processing included heating in citrate buffer for target retrieval, blocking in horse or goat serum, overnight incubation with primary antibody at 4°C and subsequent incubation with secondary antibody or Wheat Germ Agglutinin (WGA)-Alexa Fluor488 conjugate for 1 h at room temperature. Nuclei were counterstained with DAPI and the slides were mounted in Aqua Polymount. Complete sections were imaged by fluorescence microscopy using a LSM880 confocal microscope with an Airyscan module (Zeiss) at a 20X or 63X magnification. For LEPR membrane translocation studies, the super-resolution function was employed. Zen (Zeiss), ImageJ (NIH), Photoshop CS (Adobe) and Illustrator 10 (Adobe) were used for digital image processing. The membrane presentation of leptin receptor (LEPR) was evaluated by membrane co-staining with WGA. LEPR positive Schwann cells were randomly chosen per section. Per cell, five lines crossing the cell membrane were drawn in order to plot the profiles of membrane and receptor signals using the plot profile tool in ImageJ (NIH). Signal maxima of the LEPR and WGA signals were averaged per Schwann cell and peak-to-peak distances calculated. For colocalization experiments, images were taken as z-stacks. Colocalization was measured on maximum intensity projections using imageJ.

Primary antibodies: Dendra2 (1:500, polyclonal rabbit, cat.#:ABIN361314, antibodies-online.com, RRID: AB_10789591), Wheat Germ Agglutinin-Alexa Fluor 488 Conjugate (WGA, 1:200, cat.#: W11261, Invitrogen), Leptin/Obese Receptor (OBRb) IgG (1:100, monoclonal mouse, cat.#: OBR12-A, Bio Trend), S100B (EP1576Y clone, 1:200, polyclonal rabbit, cat.#: ab52642, Abcam), MBP (1:2000, polyclonal chicken, cat.#: PA1-10008, Invitrogen, RRID: AB_1077024), IBA1 (1:400, polyclonal rabbit, cat.#: 019-19741, Wako, RRID: AB_839504), SOX10 (EPR4007-104, 1:100, monoclonal rabbit, cat.#: ab180862, Abcam, RRID: AB_2721184), PCNA (1:1000, monoclonal mouse, cat.#: ab29, Abcam; RRID: AB_303394), Total OXPHOS Rodent WB Antibody Cocktail (1:1000, monoclonal mouse, cat.#: ab110413, Abcam, RRID:AB_2629281), Neurofilament H (1:1000, polyclonal chicken, cat# 822601, Bioloegend, RRID:AB_2564859), PDGFRa (1:100, polyclonal goat, cat#1062, R&D systems, RRID: AB_2236897

Secondary antibodies: Cy3-goat-anti-rabbit (1:1000, cat.#:111-165-144, Jackson Immuno, RRID:AB_2338006), Cy3-goat-anti-mouse (1:1000, cat.#:115-165-071, Jackson Immuno, RRID:AB_2338687), Alexa Fluor Plus 488 Goat anti-Chicken IgY (H+L) Cross-Adsorbed (1:1000, cat.#: A32931, Invitrogen), Alexa Fluor 488 Goat Anti-Rabbit (1:1000, cat.#: 111-545-144, Jackson Immuno, RRID:AB_2762843).

#### *In situ* hybridization

BaseScope *in situ* hybridization was performed on PFA-fixed and paraffin-embedded samples of sciatic and tibialis nerves from mice at the indicated time points. The procedure was performed using the BaseScope Detection Reagent Kit-RED (ACDbio) according to the manufacturer’s instructions. Briefly, 5 μm thick sections were cut from the embedded samples and dried over night at room temperature. After baking for 1 h at 65°C, the sections were deparaffinized with xylene and ethanol, incubated in hydrogen peroxide and subsequently in target retrieval agent for 15 min at room temperature. Sections were then incubated with protease III solution for 30 min at 40°C in the HybEZ oven (ACDbio) and subsequently hybridized with target and control probes for 2 h at 40°C. Finally, labelled samples were incubated with amplification reagents. Schwann cell counterstaining was done after washing the samples with PBS and blocking for 1 h at room temperature with goat serum in PBS/BSA. Samples were then incubated with primary anti-S100 beta (EP1576Y, 1:200, polyclonal rabbit, cat.#: ab52642, Abcam) antibody over night at 4°C. After washing with PBS, incubation with secondary antibody (Alexa Fluor 488 Goat Anti-Rabbit, 1:1000, cat.#: 111-545-144, Dianova) was performed for 1 h at room temperature. After counterstaining of nuclei with DAPI, the samples were mounted with Aqua Polymount. Images were obtained by fluorescent microscopy using a LSM880 confocal microscope with an Airyscan module (Zeiss) at a 63-fold magnification. The probe Mm-Lepr-02 (Species: mouse, mRNA accession NM_146146.3, target region: 2721-3610, cat.#: 804681, ACDbio) was used to detect *Lepr* mRNA of the long leptin receptor isoform Ob-Rb. The probe Mm-Lep (species: mouse, mRNA accession NM_008493.3, target region: 2-884, cat#: 513671, ACDbio) was used to detect leptin mRNA.

#### RNA preparation, quantitative real-time PCR (qPCR) analysis

Total RNA was extracted from indicated peripheral nerve endoneurium in a semi-automated fashion using a Maxwell RSC (Promega) and the Maxwell RSC simplyRNA Tissue Kit (Promega) according to the manufacturer’s instructions. In short, fresh frozen nerve samples were mechanically homogenized in homogenization solution using the prechilled Bead Ruptor 24 (Omni). Tissue homogenate was then mixed with 200 μl lysis buffer and loaded onto the prepared cartridge. The cartridge was then placed into the Maxwell RSC (Promega) machine and RNA further isolated and purified automatically according to the manufacturers protocol. Integrity, purity and quantity of isolated RNA were assessed using the Agilent 5200 Fragment Analyzer System (Agilent Technologies). For quantitative real-time PCR (qPCR), total RNA was reversely transcribed to cDNA using the Superscript IV Reverse Transcriptase (Thermo Fisher) and poly-Thymine together with random nonamer primer. qPCRs were performed in quadruplets using the GoTaq qPCR Master Mix (Promega) in a qTower^3^ light cycler (Analytik Jena). C_T_ values were calculated by the qPCRsoft (Analytik Jena) and transformed into fold-change expression. Values were normalized to the mean of the control group which was set to 1 and the fold-change values of experimental groups were depicted relative to the control group. *Rictor*, *Ankrd27* and *Ppia* were used as housekeeping genes, of which the geometric mean was calculated prior standardization of the genes of interest. Primer sequences are depicted below:

**Table:** 

Gene	Forward Primer	Reverse Primer
*Lep*	5’-ACATACCGCATTTCAGGGCA-3’	5’-CCCAGGTATCCCGTGTCAAC-3’
*Lepr*	5’-GTTCCAAACCCCAAGAATTG-3’	5’-GACTTCAAAGAGTGTCCGTTCTC-3’
*cJun*	5’-CCTTCTACGACGATGCCCTC-3’	5’-GGTTCAAGGTCATGCTCTGTTT-3’
*Shh*	5’-AAAGCTGACCCCTTTAGCCTA-3’	5’-TTCGGAGTTTCTTGTGATCTTCC-3’
*Pou3f1*	5’-GCGTGTCTGGTTCTGCAAC-3’	5’-AGGCGCATAAACGTCGTC-3’
*Mpz*	5’-GTCCAGTGAATGGGTCTCAGATG -3’	5’-CTTGGCATAGTGGAAAATCGAAA-3’
*Hmgcr*	5’-CAACCTTCTACCTCAGCAAGC-3’	5’-CACAGTGCCACATACAATTCG-3’
*Egr2*	5’-ACCGGGTAGAGGCTGTCA-3	5’-CAGTTCAACCCCTCTCCAAA-3
*Rnf41*	5’-TGTGACAACGCTGTGTTTGG-3’	5’-GCAGTTCATCTTTGGGCATCTC-3’
*Leprot*	5’-TTATGCTGGGATGTGCGTTG-3’	5’-TGGCAATGAAGTAGGGGATGG-3’
*Ankrd27*	5’-TCCTGCCAGTTCGAGTCCTA-3’	5’-GGCGCTGAAGGTTCTTCTGA-3’
*Rictor*	5’- ACTGACGCCAAGCAGGTTTA-3’	5’-ACATTCTTGCACCTCGGTCAT-3’
*Ppia*	5’-CACAAACGGTTCCCAGTTTT-3’	5’-TTCCCAAAGACCACATGCTT-3’
*Canx*	5’-CTTCCAGGGGATAAAGGACTTGT-3’	5’-ACATAGGCACCACCACATTCTA-3’

#### Bulk RNA sequencing

We performed sciatic nerve crush injury in *Lepr*^*fl/fl*^ (control) and *Lepr*^*fl/fl*^*::Dhh-Cre* mice and collected tissue at 2 and 4 wpc in n=4 per group, respectively. At the given time points distal ipsi- and contralateral sciatic nerve endoneuria were collected and subjected to RNA isolation and subsequent sequencing. RNA quality was assessed by measuring the RIN (RNA integrity number) using a Fragment Analyzer (Advanced Bioanalytical). Library preparation for RNA sequencing was performed using the TruSeq RNA Sample Prep Kit v2 (Illumina) starting from 200 ng of total RNA. Accurate quantification of cDNA libraries was performed by using the QuantiFluor dsDNA System (Promega). The size range of final cDNA libraries was determined by applying the DNA 1000 chip on the Fragment Analyzer (average 290–320 bp). Libraries were sequenced on the HiSeq 4000 from Illumina (SR; 1 × 50 bp; ∼30 million reads per sample).

Raw read & Quality check: Sequence images were transformed with Illumina software BaseCaller to BCL files, which was demultiplexed to fastq files with bcl2fastq v2.20. The sequencing quality was asserted using FastQC (http://www.bioinformatics.babraham.ac.uk/projects/fastqc/).

Mapping & Normalization: Sequences were aligned to the reference genome Mus musculus (GRCm38.97) using the STAR alignment tool^38^ (version 2.5) allowing for 2 mismatches within 50 bases. Subsequently, read counting was performed using featureCounts.^39^ Read counts were analyzed in the R/Bioconductor environment (version 4.0.5) using the DESeq2^41^ package version 1.31.5. Candidate genes were filtered using an absolute log_2_ fold-change >1 and FDR-corrected p-value <0.05. Gene annotation was performed using Mus musculus entries via biomaRt R package version 2.46.3.^40^

#### Protein analysis

Sciatic nerve endoneuria were mechanically homogenized in sucrose lysis buffer (320 mM sucrose, 10 mM TRIS, 1 mM NaHCO3, 1 mM MgCl2) supplemented with cOmplete Protease Inhibitor Cocktail (Roche) and PhosSTOP Phosphatase Inhibitor Cocktail (Roche) using the Bead Ruptor 24 (Omni). Samples were transferred into fresh Eppendorf tubes and protein quantification performed via A280 spectrum measurement using a NanoDrop (Thermo Fisher). Sucrose lysis buffer was used as blank and the protein concentration of each sample determined by calculating the average from at least three independent measurements. The homogenate was then diluted with sucrose lysis buffer following addition of Laemmli loading buffer (10 mM TRIS, 1.55% w/v dithiothreitol, 0.6% v/v glycerol, 0.2% w/v sodium dodecyl sulfate, 0.008% w/v bromophenol blue) to obtain a final protein concentration of 1 μg/μl in the Western blot sample. Next, the sample was vortexed and heated for 5 min at 70°C under constant shaking. 10 μg protein per sample were loaded per lane on a 4-12% Bis-Tris NuPAGE gel (Thermo Fisher) and PageRuler Plus Prestained protein Ladder (10-250kDa, Thermo Fisher) used as size control. Size separation was conducted in 3-(*N*-morpholino)propanesulfonic acid (MOPS) running buffer (25 mM MOPS, 25 mM Tris, 0.1% w/v SDS, 0.03% w/v EDTA) by electrophoresis. Subsequently, the proteins were transferred on an Amersham Hybond PVDF membrane (0.45 μm, GE Healthcare) by wet blotting in transfer buffer (48 mM TRIS, 39 mM glycerol, 10% v/v methanol) for 3 h at 100 V and 4°C. For whole protein stain, the membranes were washed 5 times for 1 min each with ddH_2_O and incubated in Fast Green FCF (Sigma Aldrich) staining solution (0.00084% w/v Fast Green, 30% v/v methanol, 6.7% v/v glacial acetic acid) for 5 min at room temperature and covered form light. Excessive Fast Green dye was removed by washing the membrane twice for 2 min each in washing solution (30% v/v methanol, 6.7% v/v glacial acetic acid) at room temperature and protected from light. Whole protein stain image was recorded on a Fusion FX Edge V.070 imager (Vilber) in fluorescence mode with an exposure time of 80 ms, an excitation wavelength of 625 nm and at an emission wavelength of 680 nm. The membrane was then washed twice for 5 min each in ddH_2_O and blocked in 5% BSA-TBST solution (5% w/v BSA, 25 mM Tris, 75 mM NaCl, 0.0005% v/v Tween 20) for 2 h at room temperature. Subsequently, the membrane was incubated in primary antibody diluted in 5% BSA-TBST overnight at 4°C under constant shaking. Following, the membrane was washed 6 times for 5 min each in TBST solution (25 mM Tris, 75 mM NaCl, 0.0005% v/v Tween 20) and incubation with secondary antibody diluted in 5% BSA-TBST was performed for 1 h at room temperature under constant shaking. After another washing for 6 times for 5 min each with TBST at room temperature, the Western Lightning Plus ECL detection solution (PerkinElmer) was prepared directly before imaging. The ECL solution was carefully poured onto the membrane and the signal detected using the Fusion FX Edge V.070 imager (Vilber) in Western Standard mode. All images were exported as uncompressed Tagged Image File Format (TIFF) files, and signal intensities were evaluated densitometrical using Fiji. All images were checked for oversaturated pixels. Relative protein abundances were only compared between samples of one blot. Relative signals from one sample were normalized to whole protein stain from the same sample.

Primary antibodies: Total OXPHOS Rodent WB Antibody Cocktail (1:1000, monoclonal mouse, cat.#: ab110413, Abcam, RRID:AB_2629281), LC3B (1:1000, polyclonal rabbit, cat.#: 2775, Cell signaling, RRID:AB_915950), p62 (SQSTM1, 1:100, polyclonal rabbit, cat.#: 5114S, Cell Signaling, RRID:AB_10624872), pERK (P-p44/42, pERK1/2, 1:2000, polyclonal rabbit, cat.#: 9101S, Cell Signaling, RRID:AB_331646), ERK (p44/42, ERK1/2, 1:2000, polyclonal rabbit, cat.#: 4695, Cell Signaling, RRID:AB_390779), HK I (C35C4, 1:1000, monoclonal rabbit, cat.#: 2024S, Cell Signaling, RRID:AB_2116996), HK II (C64G5, 1:1000, monoclonal rabbit, cat.#: 2867S, Cell Signaling, RRID:AB_2232946), pAKT (D9E) XP (Ser473, 1:1000, monoclonal rabbit, cat.#: 4060, Cell Signaling), AKT (Pan, C67E7, 1:2000, monoclonal rabbit, cat.#: 4691, Cell Signaling, RRID:AB_2617178), pSTAT3 (Tyr705) (D3A7) XP (1:1000, monoclonal rabbit, #9145, Cell signaling, RRID:AB_2491009), STAT3 (D3Z2G) (1:1000, monoclonal rabbit, #12640, Cell signaling, RRID:AB_2629499). Secondary antibodies: HRP-goat-anti-rabbit (1:5000, cat.#: 7074, Cell Signaling, RRID:AB_2099233), HRP-horse-anti-mouse (1:5000, cat.#: 7076, Cell Signaling, RRID:AB_330924).

All uncropped Western blots can be found in Data S1.

#### Autophagic flux analysis

The leptin-dependent autophagic flux was assessed by analyzing the accumulation of LC3b-II by Western blotting when lysosomal fusion is blocked. Sciatic nerve explants where cultured for six days (see ex vivo sciatic nerve culture) in the presence of the lysosomal inhibitor NH_4_Cl (15mM) and were co-treated either with or without leptin (1 μg/ml). The delta in LC3b-II flux was then calculated by dividing the densitometry value of normalized LC3b-II (relative to WPS) in the nerves treated with leptin plus the lysosomal inhibitor by the value in the control sample that was treated only with the lysosomal inhibitor.

#### Phospho explorer assay

Sciatic nerve endoneuria of five adult wildtype animals 7 days after sciatic nerve crush from ipsi- and contralateral sites were collected, pooled and freshly frozen. The samples were processed for the Phospho Explorer Antibody Microarray (Full Moon BioSystems) according to the manufacturer’s instructions while one array was used for the ipsi- and contralateral site each. Briefly, proteins were extracted from ipsi- and contralateral endoneuria and the quality and quantity assessed via A280 spectrum measurement using a NanoDrop (Thermo Fisher). Proteins were then biotinylated, conjugated to the array, and detected by a streptavidin-coupled dye. Dried arrays were send to Full Moon Biosystems for scanning and raw data generation. The generated Excel Sheet contained raw array data obtained from two spatially separated spots per epitope/antibody normalized to the median array signal. The normalized values were used to form ratios in signal intensities between paired phosphorylated and constitutive antibodies within the same experimental groups. A core analysis of the signal ratios from ipsi- and contralateral arrays was conducted via Ingenuity Pathway Analysis (IPA) software (Qiagen) using Swiss Prot Identifier for all assessed proteins. The fold-change cutoff was set to 1.5 and the p-value cutoff to 0.05 (Fisher’s Exact Test). Following, a comparative analysis between the ipsi- and contralateral sites was conducted to identify differently regulated pathways and enable upstream analysis. The comparison was performed with the recommended default settings of the software and the results were sorted by the most differentially and inversely regulated pathways and the activation Z-Score. Upstream analysis was performed with default settings from the software and the results sorted by the activation Z-Score and p value.

### Quantification and statistical analysis

Power analysis was conducted with G^∗^Power 3.1.7 before conducting *in vivo* experiments (*a priori*). An adequate power was defined as ≥ 80% (1-beta error) allowing an alpha error of 5%. If not indicated otherwise, data was processed and statistically analyzed using Microsoft Excel (Office 365) and GraphPad Prism v.7. and was expressed as mean ± standard deviation (SD). Employed statistical tests are indicated in the figure legends. Statistical differences between two groups were determined by Student’s T test, between more than two groups by one-way analysis of variance (ANOVA) using an appropriate post-hoc test, and between more than two groups and more than one time point (longitudinal) by two-way ANOVA with an appropriate post-hoc test. Before analyses, data have been tested for normal distribution in order to select parametric (normal distribution) or non-parametric (no normal distribution) testing. Statistical differences were considered to be significant when *p* < 0.05 (^∗^), *p* < 0.01 (^∗∗^), *p* < 0.001 (^∗∗∗^). All source data can be found in Data S1.

## Supplemental information

Document S1. Figures S1–S5

Data S1. Unprocessed data underlying the display items in the manuscript, related to Figures 1–7 and S1–S5, in which all the relevant figures are listed

Document S2. Article plus supplemental information
