# Supplementary material for: Adipo-glial signaling mediates metabolic adaptation in peripheral nerve regeneration
Source: Cell Metab. 2023 Dec 5;35(12):2136–2152.e9. doi: 10.1016/j.cmet.2023.10.017 (PMC10722468; doi:10.1016/j.cmet.2023.10.017)
Supplement: Document S1. Figures S1–S5 [file mmc1.pdf]

**Cell Metabolism, Volume *35***

# Supplemental information

# Adipo-glial signaling mediates metabolic adaptation in peripheral nerve regeneration

**Venkat Krishnan Sundaram, Vlad Schütza, Nele H. Schröter, Aline Backhaus, Annika Bilsing, Lisa Joneck, Anna Seelbach, Clara Mutschler, Jose A. Gomez-Sanchez, Erik Schäffner, Eva Ernst Sánchez, Dagmar Akkermann, Christina Paul, Nancy Schwagarus, Silvana Müller, Angela Odle, Gwen Childs, David Ewers, Theresa Kungl, Maren Sitte, Gabriela Salinas, Michael W. Sereda, Klaus-Armin Nave, Markus H. Schwab, Mario Ost, Peter Arthur-Farraj, Ruth M. Stassart, and Robert Fledrich**

# Supplemental Figure 1:

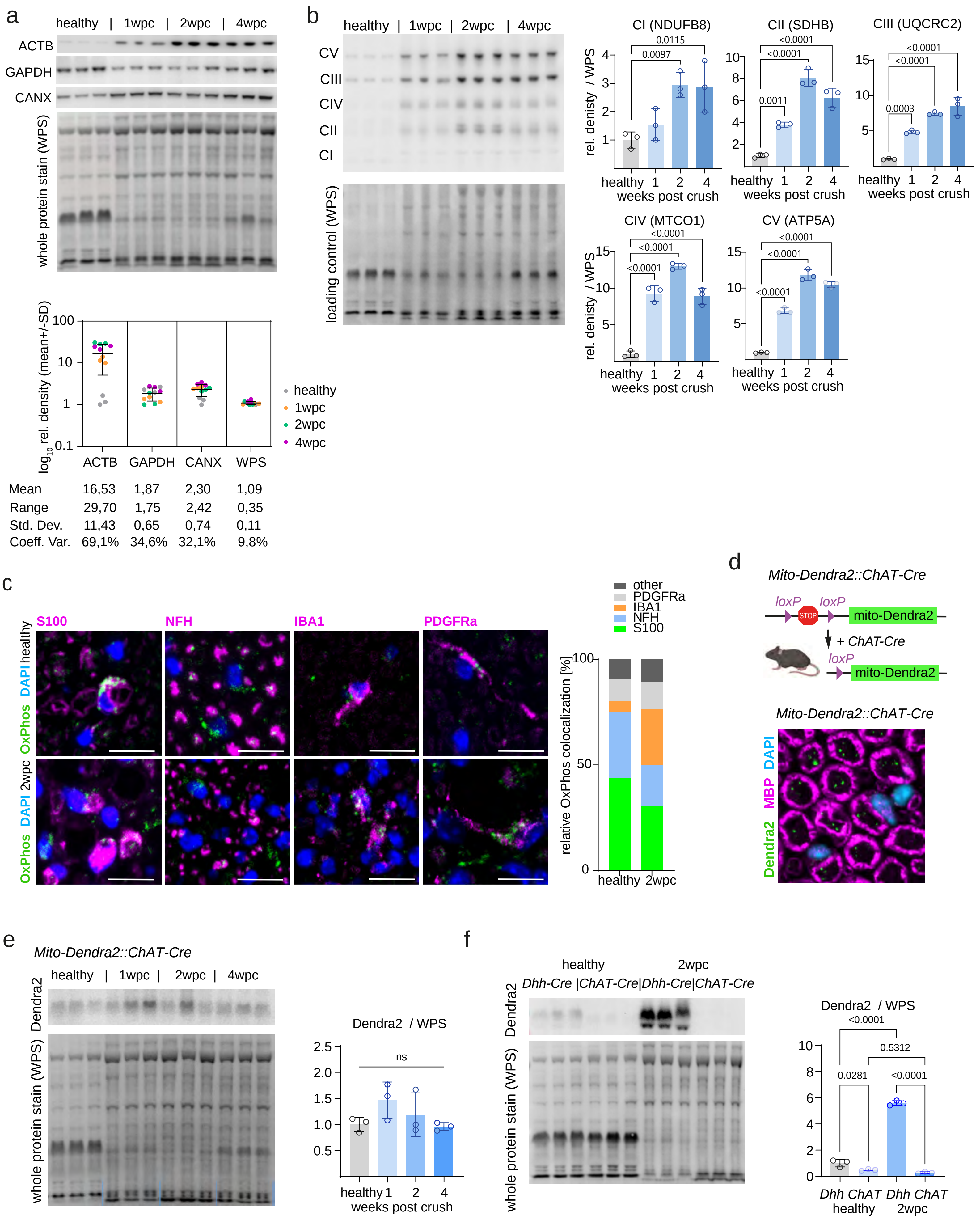


| | ACTB | GAPDH | CANX | WPS |
|---|---|---|---|---|
| Mean | 16,53 | 1,87 | 2,30 | 1,09 |
| Range | 29,70 | 1,75 | 2,42 | 0,35 |
| Std. Dev. | 11,43 | 0,65 | 0,74 | 0,11 |
| Coeff. Var. | 69,1% | 34,6% | 32,1% | 9,8% |

**Supplemental Figure 1: Dynamic regulation of mitochondrial protein expression in peripheral nerves after injury, related to Figure 1.**

**a.** Determination of the least variable loading control in Western blotting of sciatic nerve protein lysates in healthy nerves and at different time points post crush (1, 2, 4 weeks post crush). Three conventionel loading control proteins, ACTB, GAPDH and CANX and a whole protein membrane stain (WPS) were tested (top panel) and the densitometric analysis revealed WPS as the most stable loading control (bottom panel, n=3 per group).

**b.** The expression of oxidative phosphorylation complexes (CI to CV) is induced in injured nerves as revealed by Western blot analyses of distal sciatic nerve endoneurial protein lysates after 1,2 and 4 wpc. An antibody mix against proteins of all five complexes of the respiratory chain was used (left, CI-CV). Individual bands were quantified relative to whole protein stain (WPS) as loading control (right, one-way ANOVA with Dunnett's post test).

**c.** Representative immunohistochemical images of OxPhos colocalization with different cell type markers (S100 for Schwann cells, NFH for axons, IBA1 for macrophages, PDGFRa for fibroblasts) in healthy and 2wpc sciatic nerve cross sections (left, OxPhos in green, cell type markers in magenta, nuclei DAPI in blue). Relative OxPhos colocalization in different cell types is quantified and the respective proportions are expressed as parts of a whole (100%, analysis was performed in n=3-4 animals, respectively, and the mean values calculated).

**d.** Schematic presentation of the genetic strategy to generate axon-specific mitochondria reporter in mice. *Stop-flox-mito-Dendra2* mice were crossbred with *ChAT-Cre* driver mice to selectively induce fluorescently Dendra2-labeled mitochondria in cholinergic neurons and their axons (top). Sciatic nerve cross section from a reporter mouse confirms Dendra2 fluorescence selectively inside myelin rings (MBP, magenta) in axons (bottom, Dendra2 in green, myelin MBP in magenta, nuclei DAPI in blue).

**e.** Western blot analyses of distal sciatic nerve endoneurial protein lysates from motor axonal mitochondrial reporter mice from **d.** at 1,2 and 4 wpc and in healthy nerves revealed low baseline abundance of Dendra2 and only a minor non-significant dynamics post crush (n=3 per group, one-way ANOVA with Tukey's post test).

**f.** Western blot analyses of Dendra2 expression in distal sciatic nerve protein lysates from axonal (*ChAT*-Cre) and Schwann cell (*Dhh*-Cre) mitochondrial reporter mice at 2wpc and in healthy nerves. The densitometric analysis revealed a drastically low abundance of Dendra2 in the *ChAT*-Cre mice when compared to the *Dhh*-Cre mice at 2wpc (n=3 per group, one-way ANOVA with Tukey's post test).

Supplemental Figure 2:

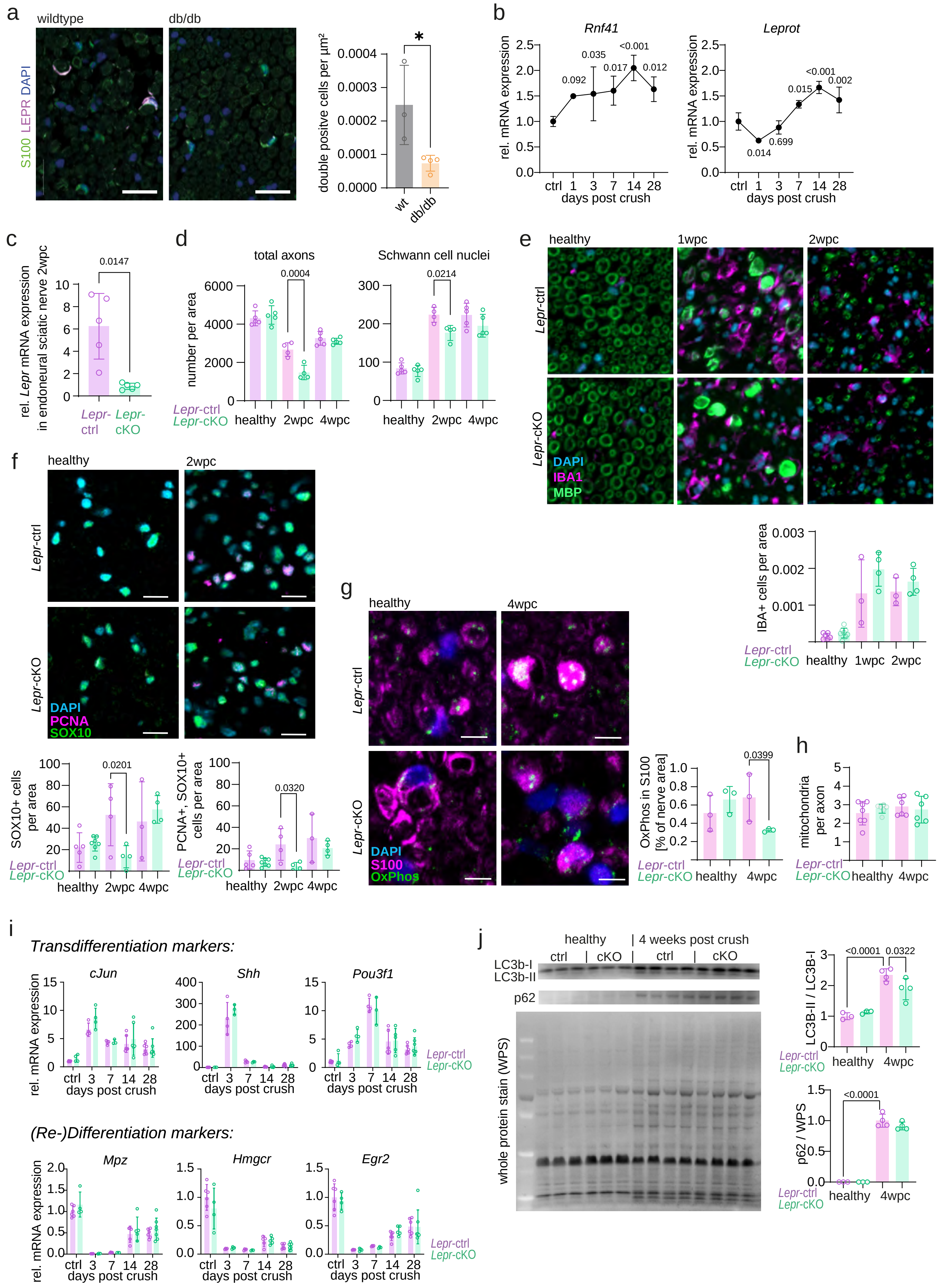

**Supplemental Figure 2: The ablation of *Lepr* from Schwann cells impacts nerve repair, related to Figure 2 and Figure 3.**

**a.** Representative IHC images of LEPR expression in Schwann cells (S100) between WT animals and *db/db* animals. Quantification revealed a reduction of LEPR positive SC cells in the *db/db* mice. (n=3 to 4 per group, student's t-test).

**b.** Relative mRNA expression timeline of *Rnf41* and *Leprot* in WT nerves at the indicated time points post crush injury.

**c.** Relative *Lepr* mRNA expression measured by qPCR in sciatic nerve endoneuria of injured *Lepr*-ctrl mice and Schwann cell specific *Lepr*-cKO mice at two weeks post crush (2wpc) revealed a reduction of *Lepr* mRNA expression in *Lepr*-cKO mice (n=5 per group, one-way ANOVA with Tukey's post test).

**d.** Electron microscopic quantification from **Fig. 3e** of total axons (left) and Schwann cell nuclei (right) per area in tibial nerve cross sections from adult control (*Lepr*-ctrl) and Schwann cell *Lepr* knockout (*Lepr*-cKO) mice before (healthy) and two and four weeks after crush (2wpc, 4wpc; n = 4-5 per group, one-way ANOVA with Tukey's post-hoc test).

**e.** No alteration in macrophage abundance in injured nerves of *Lepr*-cKO mice compared to controls. Immunohistochemical quantification of IBA1 positive macrophages (magenta) in sciatic nerve cross sections in healthy nerves and at 1 and 2 wpc in *Lepr*-ctrl and Schwann cell specific *Lepr*-cKO mice. Myelin is counterstained with MBP (green), and nuclei with DAPI (blue, n=4-7 per group, one-way ANOVA with Tukey's post test).

**f.** Transiently impaired Schwann cell proliferation of *Lepr*-cKO mice after nerve injury. Immunohistochemical quantification of SOX10 (green) positive Schwann cells and proliferating PCNA (magenta) and SOX10 (green) double positive Schwann cell nuclei (DAPI, blue) per area in sciatic nerve cross sections of *Lepr*-ctrl and Schwann cell specific *Lepr*-cKO mice. Top panels display example pictures for healthy control and 2wpc cross sections (scale: 10µm), bottom panels show quantification including the 4wpc time point (n=4-7 per group, one-way ANOVA with Tukey's post test).

**g.** Representative immunohistochemical images of OxPhos colocalization with S100 (Schwann cells) in healthy and 4wpc sciatic nerve cross sections of *Lepr*-ctrl and *Lepr*-cKO mice (left, OxPhos in green, S100 in magenta, nuclei DAPI in blue) revealed less OxPhos immunoreactivity in mutant Schwann cells compared to controls at 4wpc. OxPhos in S100 is normalized to the total area of the section (expressed as percentage, right).

**h.** Quantification of axonal mitochondria from **Fig. 3e.** shows no difference in axonal mitochondria numbers between *Lepr*-ctrl and *Lepr*-cKO animals in healthy nerves and at 4wpc.

**i.** No alteration in Schwann cell differentiation marker expression in injured nerves of *Lepr*-cKO mice compared to controls. Relative mRNA expression of transdifferentiation (top panel) and redifferentiation (bottom panel) marker in sciatic nerve endoneuria of adult control (*Lepr*-ctrl) and Schwann cell *Lepr* knockout (*Lepr*-cKO) mice at different days post crush. Contralateral sciatic nerve endoneuria were used as control (n = 3-5 per group, one-way ANOVA with Tukey's post hoc test).

**j.** Western blot analysis (left) and densitometrical quantification (right) of LC3b-I and II and p62 protein abundances in sciatic nerve endoneurium lysates of contra- (healthy) and ipsilateral sites from adult control (*Lepr*-ctrl) and Schwann cell *Lepr* knockout (*Lepr*-cKO) mice at 4wpc. Whole protein stain (WPS) was performed as loading control for p62 (n = 3-4 per group, one-way ANOVA with Tukey's post-hoc test).

Supplemental Figure 3:

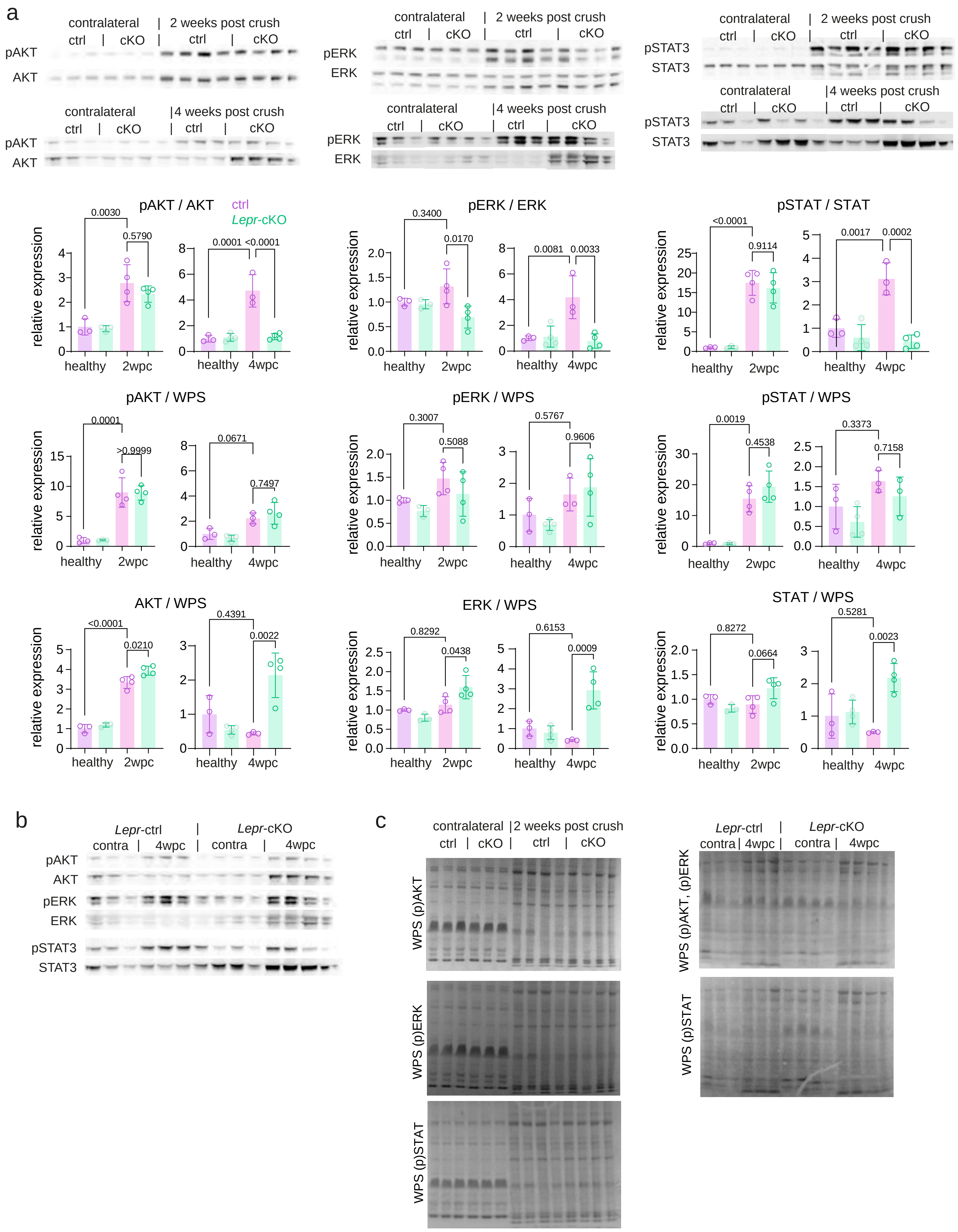

**Supplemental Figure 3: Analyses of LEPR downstream signalling cascades, related to Figure 3 and Figure 4.**

**a.** Altered expression of leptin receptor downstream signaling proteins in injured nerves of *Lepr*-cKO mice. Western blot analyses (top panels) and respective densitometrical quantification (bottom panels) of pAKT / AKT, pERK / ERK and pSTAT3 / STAT3 protein abundance ratios and respective constitutive expression values (normalized to WPS, respectively) in crushed sciatic nerve endoneurium lysates from adult control (*Lepr*-ctrl) and Schwann cell *Lepr* knockout (*Lepr*-cKO) mice at 2 and 4wpc (n = 3-4 per group, one-way ANOVA with Tukey's post-hoc test). Western blots at 4wpc were cropped and re-aligned to match the order of 2wpc blots for better comparison.

**b.** Original non-cropped 4wpc Western blots are shown.

**c.** Whole protein stain (WPS) of all the blots.

Supplemental Figure 4:

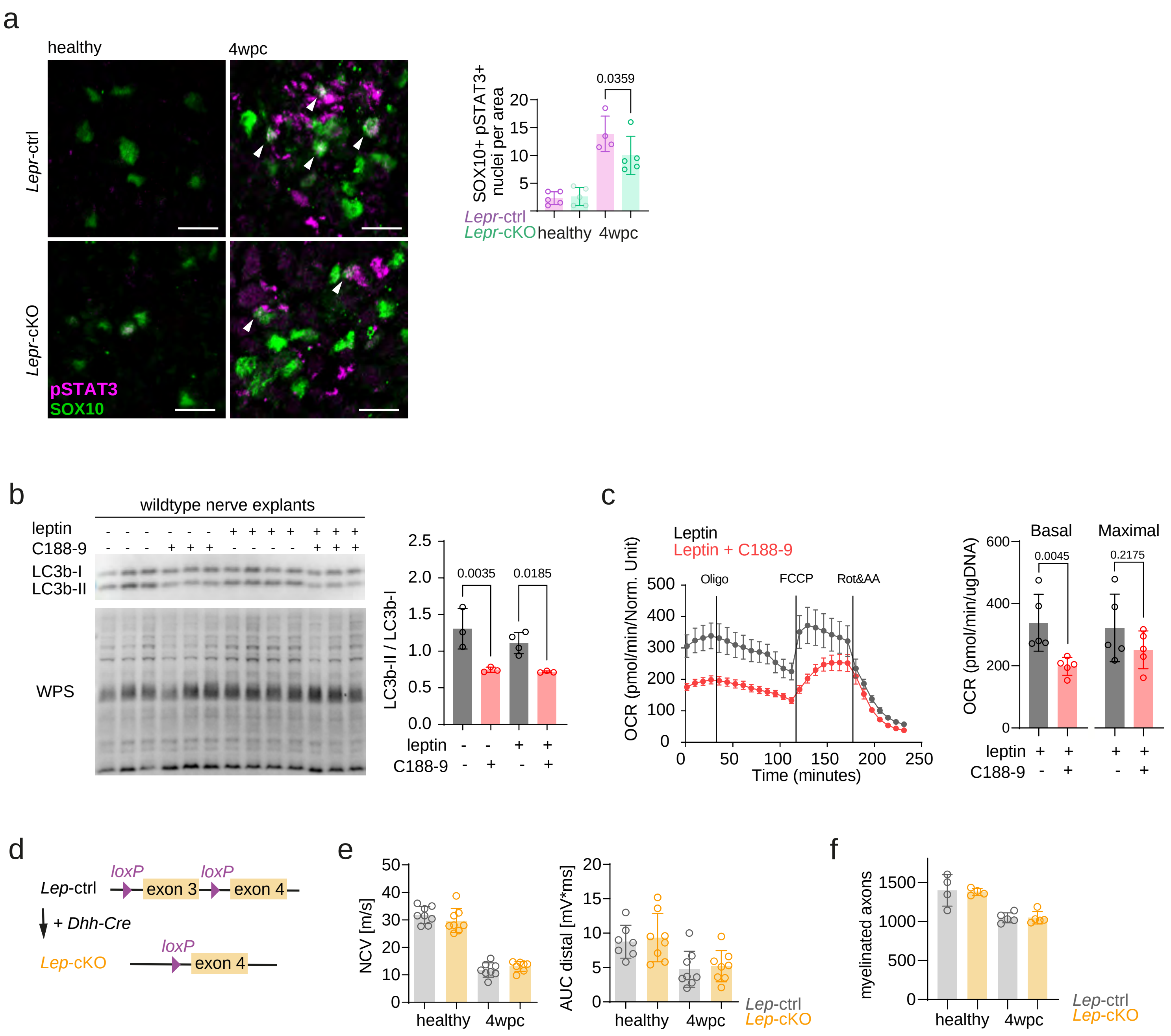

**Supplemental Figure 4: The role of STAT3 dowstream of leptin signaling, related to Figure 4 and Figure 5.**

**a.** Immunohistochemical quantification of pSTAT3 (magenta) positiv SOX10 (green) positive Schwann cells per area in sciatic nerve cross sections of *Lepr*-ctrl and *Lepr*-cKO mice in healthy nerves and at 4wpc. Left panels display example pictures (scale: 10µm), right panel shows quantification (n=4-5 per group, one-way ANOVA with Tukey's post test).

**b.** Schematic representation of the genetic strategy for the generation of conditional Schwann cell *Lep* knockout mice. *Lep-flox* mice (*Lep*-ctrl) were crossbred with *Dhh-Cre* driver mice to ablate exon 3 of the *Lep* gene specifically in Schwann cells (*Lep*-cKO).

**c.** Functional repair is not altered in *Lep*-cKO mice as revealed by electrophysiology. NCV (left) and CMAP area under the curve (AUC, right) from contra- (healthy) and ipsilateral sites (4wpc) from adult control (*Lep*-ctrl) and Schwann cell *Lep* knockout (*Lep*-cKO) mice at 4wpc (n=8 per group, one-way ANOVA with Tukey's post-hoc test).

**d.** Number of myelinated axons in tibial nerve semi-thin cross sections from contra- (healthy) and ipsilateral sites (4wpc) from adult control (*Lep*-ctrl) and Schwann cell *Lep* knockout (*Lep*-cKO) mice at 4wpc reveales no difference between genotypes (n = 4-5 per group, one-way ANOVA with Tukey's post-hoc test).

**e.** Western blot analysis (left) and densitometrical quantification (right) of LC3b-I and II in WT *ex vivo* sciatic nerve explants at 6div treated with leptin or with leptin and STAT3 inhibitor (C188-9). Whole protein stain (WPS) was used as loading control (n = 3-4 per group, one-way ANOVA with Tukey's post-hoc test).

**f.** Seahorse respirometry and measurement of the oxygen consumption rate (OCR) in WT nerve explants after six days *ex vivo* (6div). Explants were either treated with leptin (grey) or with Leptin and C188-9 (red). OCR traces are depicted on top and quantification of the basal and maximal OCR is shown at the bottom. OCR was normalized to the gDNA content (n=4-5 per group, student's t-test).

Supplemental Figure 5:

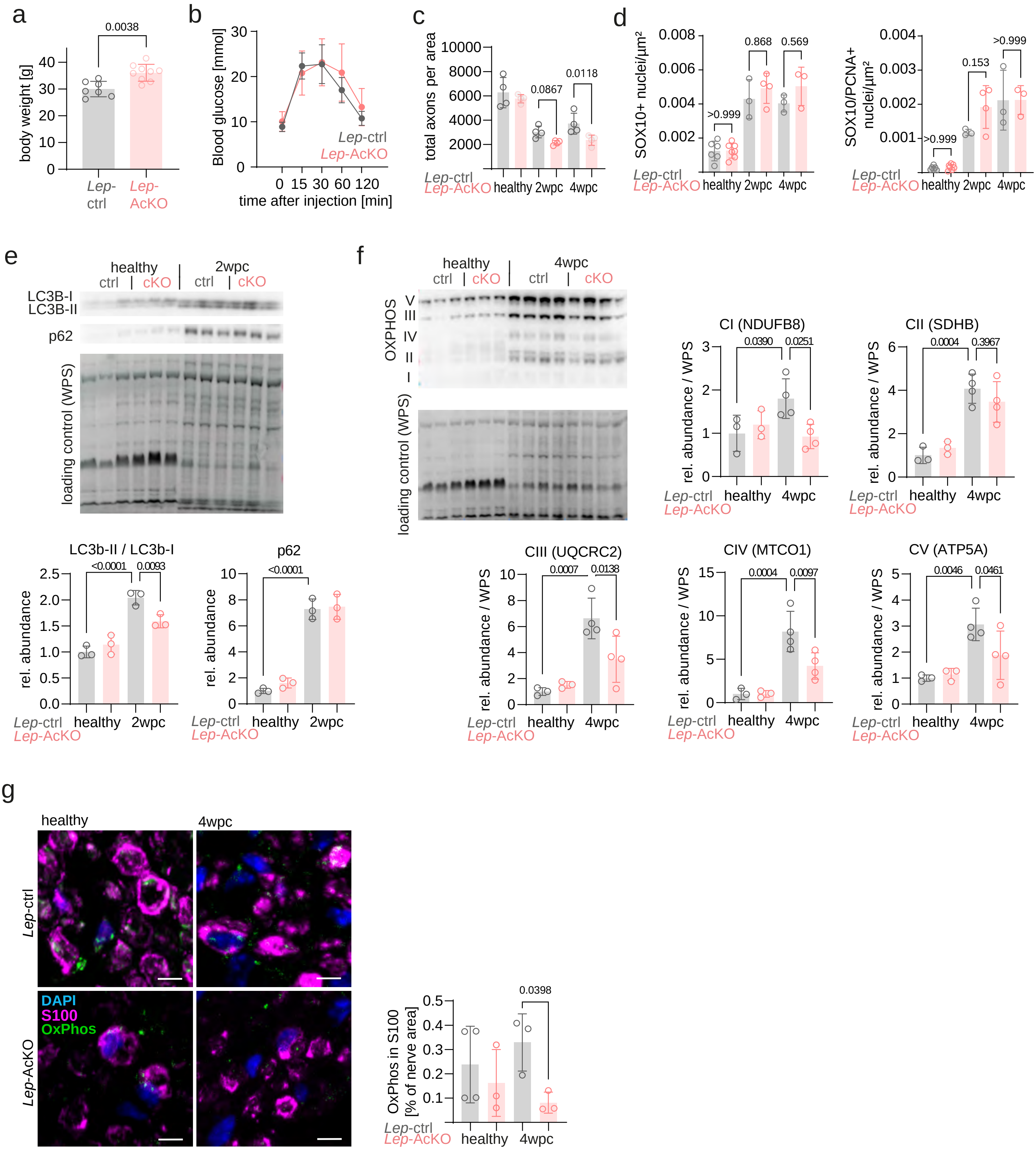

**Supplemental Figure 5: Leptin ablation from adipocytes impairs nerve repair, related to Figure 6.**

**a**. Body weight measurements revealed slight increase in adipocyte *Lep* knockout (*Lep*-AcKO) mice compared to control (*Lep*-ctrl) at 4wpc, i.e. six weeks after recombination (n = 10 per group, Student's t-test).

**b.** Glucose tolerance test of adult control (*Lep*-ctrl) and adipocyte *Lep* knockout (*Lep*-AcKO) mice revealed no difference between groups at 4wpc. Shown are blood glucose levels at indicated time points after intraperitoneal glucose injection (n = 10 per group, two-way ANOVA).

**c.** Quantification of total axons per area in electron micrographs from **Fig. 6j** in tibial nerve cross sections from adult control (*Lep*-ctrl) and induced adipocyte *Lep* knockout (*Lep*-AcKO) mice before (healthy) and two and four weeks after crush (2wpc, 4wpc; n = 4-5 per group, one-way ANOVA with Tukey's post-hoc test).

**d.** Quantification of SOX10+ nuclei (left) and SOX10+PCNA+ nuclei (right) per unit area in tibial nerve cross sections from adult control (*Lep*-ctrl) and induced adipocyte *Lep* knockout (*Lep*-AcKO) mice before (healthy) and two and four weeks after crush (2wpc, 4wpc; n = 3-4 per group, one-way ANOVA with Tukey's post-hoc test).

**e.** Western blot analysis (top panels) and densitometrical quantification (bottom panels) of autophagy markers in sciatic nerve endoneurium lysates of contra- (healthy) and injured sides from adult *Lep*-ctrl and adipocyte *Lep*-AcKO mice at 2wpc. The ratio of LC3b-II over LC3b-I was calculated and shows a decrease in *Lep*-cKO mice compared to controls at 2wpc. For the p62 autophagy substrate (protein abundance was normalized to whole protein stain, WPS, as loading control), Lepr-cKO show

**f.** After injury, the increased expression of proteins related to mitochondrial respiration as seen in nerve endoneuria of controls, is reduced in adipocyte *Lep*-AcKO mice at 4wpc. Western blot analyses (left panels) and densitometric quantifications (right panels) of oxidative phosphorylation protein complexes I – V (OXPHOS I – V) protein abundances in sciatic nerve endoneurium lysates of contra- (healthy) and injured (4wpc) sides from adult *Lep*-ctrl and *Lep*-AcKO mice (n = 3–4 per group, one-way ANOVA with Tukey's post-hoc test. Whole protein stain (WPS) were used as loading controls.

**g.** Representative immunohistochemical images of OxPhos colocalization with S100 positive Schwann cells in healthy and 4wpc sciatic nerve cross sections of *Lep*-ctrl and *Lep*-AcKO mice (left, OxPhos in green, S100 in magenta, nuclei DAPI in blue). OxPhos in S100 is normalized to the total nerve area and is expressed as percentage (right). IHC quantification revealed that OxPhos occupancy in S100+ Schwann cells is lesser at 4wpc in *Lep*-AcKO mice.
