# Supplementary material for: Adipo-glial signaling mediates metabolic adaptation in peripheral nerve regeneration
Source: Cell Metab. 2023 Dec 5;35(12):2136–2152.e9. doi: 10.1016/j.cmet.2023.10.017 (PMC10722468; doi:10.1016/j.cmet.2023.10.017)

Figure 1d

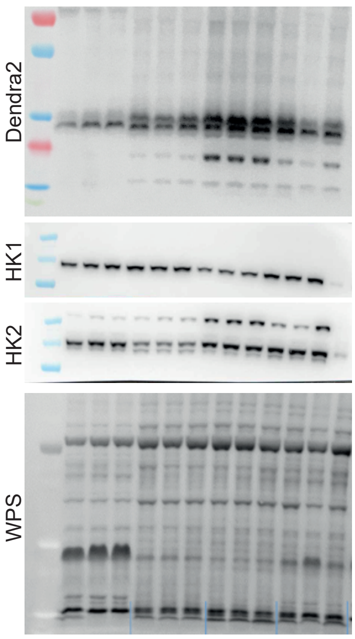

Figure 3g

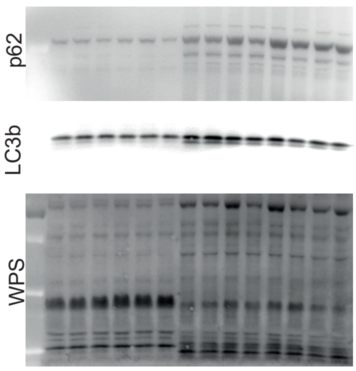

Figure 4c

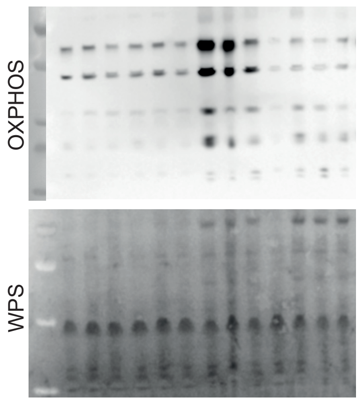

Figure 5c

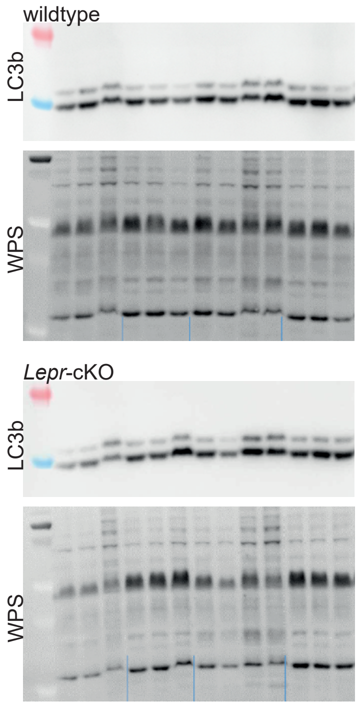

Suppl. Fig. 1a

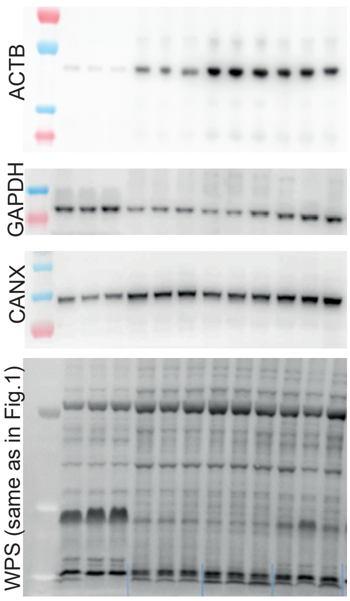

Suppl. Fig. 1b

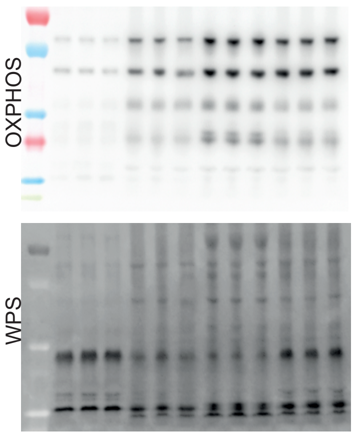

Suppl. Fig. 1e

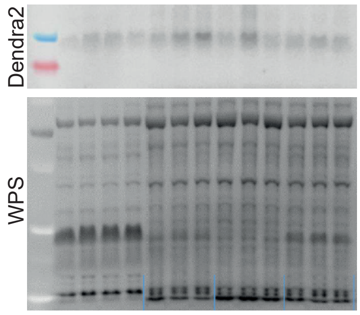

Suppl. Fig. 1f

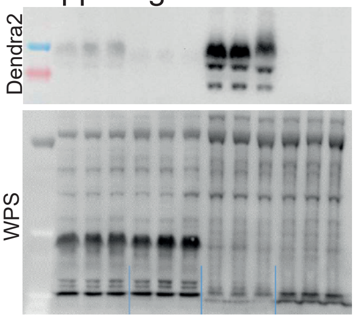

Suppl. Fig. 2j

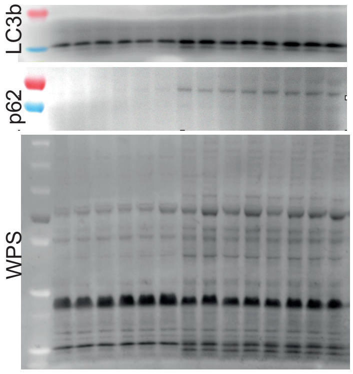

Suppl. Fig. 5e

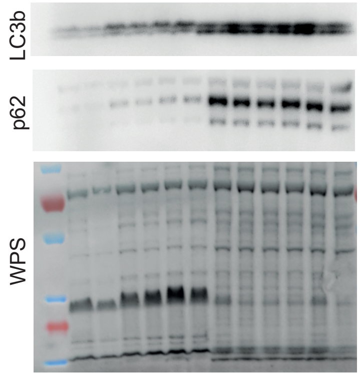

Suppl. Fig. 3a-c

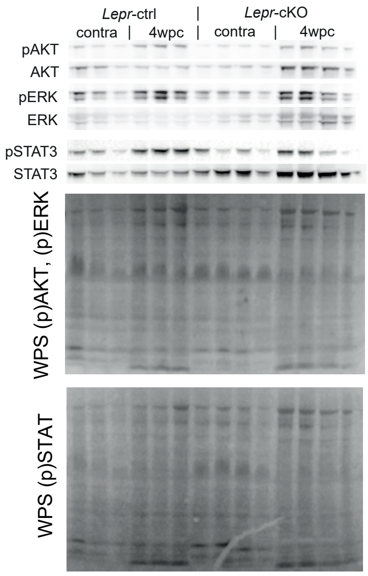

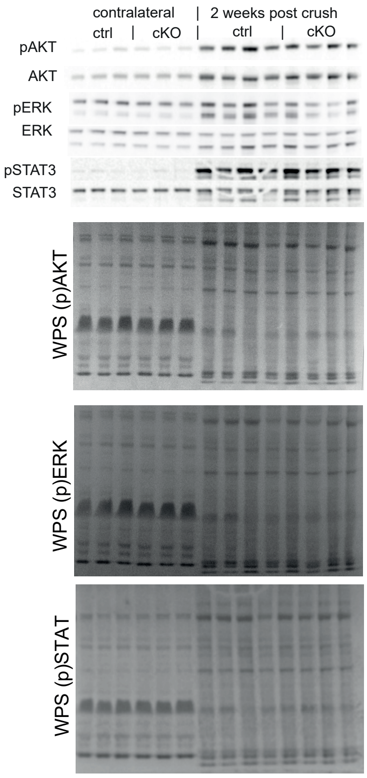

Suppl. Fig. 4e

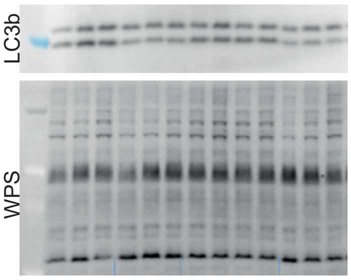

Suppl. Fig. 5f

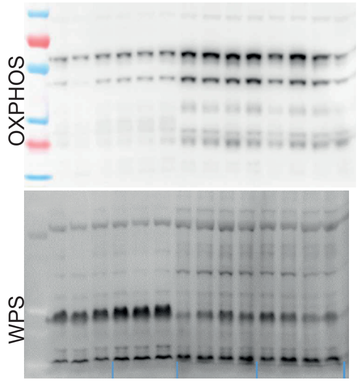

Supplement: Data S1. Unprocessed data underlying the display items in the manuscript, related to Figures 1–7 and S1–S5, in which all the relevant figures are listed [file mmc2.zip › SuppMaterial_uncroppedWBs.pdf]
